# Antitumor profile of the PI3K inhibitor ZSTK474 in human sarcoma cell lines

**DOI:** 10.18632/oncotarget.26216

**Published:** 2018-10-12

**Authors:** Nachi Namatame, Naomi Tamaki, Yuya Yoshizawa, Mutsumi Okamura, Yumiko Nishimura, Kanami Yamazaki, Miwa Tanaka, Takuro Nakamura, Kentaro Semba, Takao Yamori, Shin-ichi Yaguchi, Shingo Dan

**Affiliations:** ^1^ Division of Molecular Pharmacology, Cancer Chemotherapy Center, Japanese Foundation for Cancer Research, Tokyo, Japan; ^2^ R&D Center, Zenyaku Kogyo Co. Ltd, Tokyo, Japan; ^3^ Division of Carcinogenesis, Cancer Institute, Japanese Foundation for Cancer Research, Tokyo, Japan; ^4^ Department of Life Science and Medical Bioscience, School of Advanced Science and Engineering, Waseda University, Tokyo, Japan; ^5^ Present address: Center for Product Evaluation, Pharmaceuticals and Medical Devices Agency, Tokyo, Japan

**Keywords:** sarcoma, PI3K, anticancer agent, cell line panel, oncogenic chromosomal translocation

## Abstract

Treatment of patients with advanced sarcoma remains challenging due to lack of effective medicine, with the development of novel drugs being of keen interest. A pan-PI3K inhibitor, ZSTK474, has been evaluated in clinical trials against a range of advanced solid tumors, with clinical benefit shown in sarcoma patients. In the present study, we developed a panel of 14 human sarcoma cell lines and investigated the antitumor effect of 24 anticancer agents including ZSTK474, other PI3K inhibitors, and those clinically used for sarcoma treatment. ZSTK474 exhibited a similar antiproliferative profile to other PI3K inhibitors but was clearly different from the other drugs examined. Indeed, ZSTK474 inhibited PI3K-downstream pathways, in parallel to growth inhibition, in all cell lines examined, showing proof-of-concept of PI3K inhibition. In addition, ZSTK474 induced apoptosis selectively in Ewing's sarcoma (RD-ES and A673), alveolar rhabdomyosarcoma (SJCRH30) and synovial sarcoma (SYO-1, Aska-SS and Yamato-SS) cell lines, all of which harbor chromosomal translocation and resulting oncogenic fusion genes, *EWSR1-FLI1*, *PAX3-FOXO1* and *SS18-SSX*, respectively. Finally, animal experiments confirmed the antitumor activity of ZSTK474 *in vivo*, with superior efficacy observed in translocation-positive cells. These results suggest that ZSTK474 could be a promising drug candidate for treating sarcomas, especially those harboring chromosomal translocation.

## INTRODUCTION

Sarcomas are rare malignant tumors of mesenchymal origin, such as muscle, fat, bone and other connective tissues, with > 50 different subtypes reported to date [1]. It is estimated about 13,000 cases of soft tissue and bone sarcoma are diagnosed per year in the United States [2, 3], with morbidity due to sarcomas found to be higher in children and young adults than in adults. There are two genetically characterized groups in sarcomas [4, 5]; one is characterized by a specific chromosomal translocation event on a background of relatively few other chromosomal changes, which is represented by Ewing's sarcoma (ES) accompanied by the fusion of the *EWSR1* (Ewing sarcoma region 1, also called *EWS*) gene and one of the ETS transcriptional factor genes (*FLI1* or *ERG*) [6] and synovial sarcoma (SS) accompanied by the fusion of the *SS18* (Synovial Sarcoma Translocation, Chromosome 18, also called *SYT*) gene and one of the *SSX* (Synovial Sarcoma, X Breakpoint) genes (*SSX1*, *SSX2* or *SSX4*) [7–9]. These fusion gene products have a strong transformation activity, with the growth and survival of the cells containing these become exclusively dependent on the particular fusion [10–12]. Another group is consisted of sarcomas harboring complex karyotypic abnormalities, accompanied by multiple gene mutations, gene amplifications and chromosomal aberrations, which frequently include the impairment of cell cycle checkpoint genes [13].

Although several animal models and patient-derived xenograft models have been established to understand the molecular mechanism underlying sarcoma development, few therapeutics options are available thus far due to its complex character [4]. The primary approach for treating sarcomas is surgical resection, while systemic therapy is needed for patients with inoperable sarcoma or with metastasis. For chemosensitive sarcomas which are histologically diagnosed as small round cell sarcomas, doxorubicin and ifosfamide are applicable; however, the effectiveness of such treatments are limited [14, 15]. In the last decade, novel targeted agents have been developed to specifically address particular biological mechanisms identified for distinct subsets of sarcoma [13, 16]. Pazopanib was the first molecularly targeted agent approved for sarcoma treatment on the basis of modest benefit shown in the PALETTE study [17], with other small molecule inhibitors being examined in both preclinical and early phase clinical trials [18–21].

We previously established a panel of 39 human cancer cell lines (JFCR39) composed of various carcinoma types [22–24]. The JFCR39 panel, as well as the NCI60 set developed by the National Cancer Institute [25], has been used as an *in vitro* tool to identify novel antitumor agents and predict modes of action, as well as to identify predictive biomarkers relating to antitumor efficacy. “*Fingerprints*” are defined as the patterns of differential drug efficacy across the panel of cell lines, and have been found to be reflective of mode of action. Indeed, the mode of action of novel anticancer compounds could be predicted by correlation analysis of its fingerprint to those of reference compounds *via* a bioinformatic approach called *COMPARE* [26]. Using this system, we previously identified a novel phosphatidylinositol-3 kinase (PI3K) inhibitor, ZSTK474, by similarity to a known PI3K inhibitor, LY294002 [27]. This compound has been shown to exert a broad spectrum of antitumour activity across the panel of cell lines tested *in vitro* and *in vivo* [28–30]. Clinical trials of ZSTK474 performed in the U.S.A. revealed that it was well-tolerated, with nine of the 39 recipients exhibiting stable disease (SD) lasting for > eight weeks of which four of these, including three sarcoma patients, had SD for an extended period (for >16 weeks) [31]. Interestingly, there were four sarcoma recipients in the overall cohort and three of these were included in the prolonged SD group, suggesting that ZSTK474 could be useful in sarcoma therapy. We had previously been studying at a preclinical level the antitumor effect of ZSTK474 against various carcinoma cell lines derived from different organs, albeit not sarcoma cell lines. The above-mentioned clinical trial results prompted us to examine the antitumor profile of ZSTK474 in sarcoma cell lines from various origins in preclinical models.

In the present study, we characterized the antitumor profile of ZSTK474 in sarcoma cells *via* the use of a cell line panel approach, akin to JFCR39. We collected 14 commercially-available sarcoma cell lines from various origins and established a sarcoma panel. A total of 24 anticancer agents including ZSTK474, other PI3K inhibitors, and those clinically used for sarcoma treatment were examined with respect to their antitumor profiles across the panel of sarcoma cell lines in terms of effects on tumor growth, PI3K-downstream signaling pathway alterations and apoptosis induction *in vitro* and *in vivo*. These profiles were then compared to genetic backgrounds of sarcoma cells in an attempt to identify candidate predictive markers for the antitumor efficacy of ZSTK474.

## RESULTS

### Characterization of gene mutations and activation status of signaling proteins in the sarcoma cell line panel

We first examined the genetic background of 14 cell lines within the sarcoma panel by amplicon sequencing. The origin and mutation status of each of the cell lines is shown in Table 1. Of note, eleven of the 14 cell lines had mutations in *TP53*, which was the most frequently mutated gene among the genes examined. In addition, gain of function mutations, which gave new activity or enhancement of its activity to the proteins, were commonly found in *KIT* (M541L, four cell lines), *BRAF* (V600E, three cell lines) and *NRAS* (Q61K/H, two cell lines) genes. In contrast, none of the cell lines in this panel harbored known gain of function mutations in the *PIK3CA* gene at the hotspot residues (E542, E545 and H1047). Missense mutations were not observed in the *PTEN* gene in these cell lines, while intronic deletions were observed in the HT-1080, RD and RD-ES cell lines.

**Table 1:** Panel of 14 sarcoma cell lines and their molecular profile determined by amplicon sequence

Cell line	Reported mutations/fusion genes	Gene mutations detected by amplicon sequence
Fibrosarcoma		
HT-1080	*CDKN2A* (*p.0?*)	*IDH1* (R132C), *NRAS* (Q61K), *PDGFRA* (S566_E571>K), *PTEN* (*p.?*), *TP53* (G105fs^*^18), *HRAS* (H27H)
SW684		*APC* (E1494fs^*^19), *CDKN2A* (P114L), *TP53* (R213^*^, R120^*^, R81^*^, G105fs^*^18, R342fs^*^3, R213fs^*^34, R342fs^*^3)
Giant cell sarcoma		
GCT	*CDKN2A* (L32R), *TP53* (Q317^*^)	*BRAF* (V600E), *JAK3* (V221I), *TP53* (R248W, N247N, R155W), *HRAS* (H27H)
Leiomyosarcoma		
SK-UT-1		*APC* (Q1096^*^), *PIK3CA* (R88Q), *SMARCB1* (*p.?*), *TP53* (R175H, R248Q, R82H, R43H, R155Q), *VHL* (L128fs^*^31), *HRAS* (H27H)
Rhabdomyosarcoma		
SJCRH30 (alveolar)	*PAX3-FOXO1*	*KIT* (M541L), *PDGFRA* (V824V, S566_E571>K), *TP53* (R273C, R280S, Y205C)
RD (embryonic)		*NRAS* (Q61H), *KIT* (M541L), *PTEN* (*p.?*), *HRAS* (H27H), *TP53* (G105fs^*^18, R248fs^*^97, M246_P250delMNRRP, R248W, R155W)
Osteosarcoma		
HOS	*CDKN2A* (*p.0?*)	*TP53* (R156R, V157fs^*^13), *PDGFRA* (S566_E571>K), *HRAS* (H27H)
KHOS-240S		*TP53* (V157fs^*^13, R156P), *HRAS* (H27H)
Saos-2	*RB1* (*p.0?*), *TP53* (*p.0?*)	*PDGFRA* (S566_E571>K)
Liposarcoma		
SW872	*PTEN* (*p.0?*)	*APC* (E1494fs^*^19), *BRAF* (V600E), *CDKN2A* (P135L, R80^*^), *PDGFRA* (V824V, S566_E571>K), *TP53* (T253A, I251del, I251N, I251_T253delIL)
Synovial sarcoma		
SW982	no *SS18-SSX* mutation was detected	*BRAF* (V600E), *PDGFRA* (S566_E571>R)
Chondrosarcoma		
SW1353		*CDKN2A* (*p.?*), *IDH2* (R172S), *KIT* (M541L), *KRAS* (G12V), *SMARCB1*(*p.?*), *TP53* (V203L, V157G)
Uterine sarcoma		
MES-SA		*KIT* (M541L, K546K), *HRAS* (H27H), *CDKN2A* (*p.?*)
Ewing's sarcoma		
RD-ES	*EWSR1-FLI1*	*APC* (E1494fs^*^19), *PDGFRA* (S566_E571>K), *TP53* (R273C), *SMARCB1* (*p.?*), *HRAS* (H27H)

Footnote: *p.0?*: probably no protein is produced; *p.?*: protein has not been analyzed, an effect is expected but difficult to predict.

**Figure 1:**
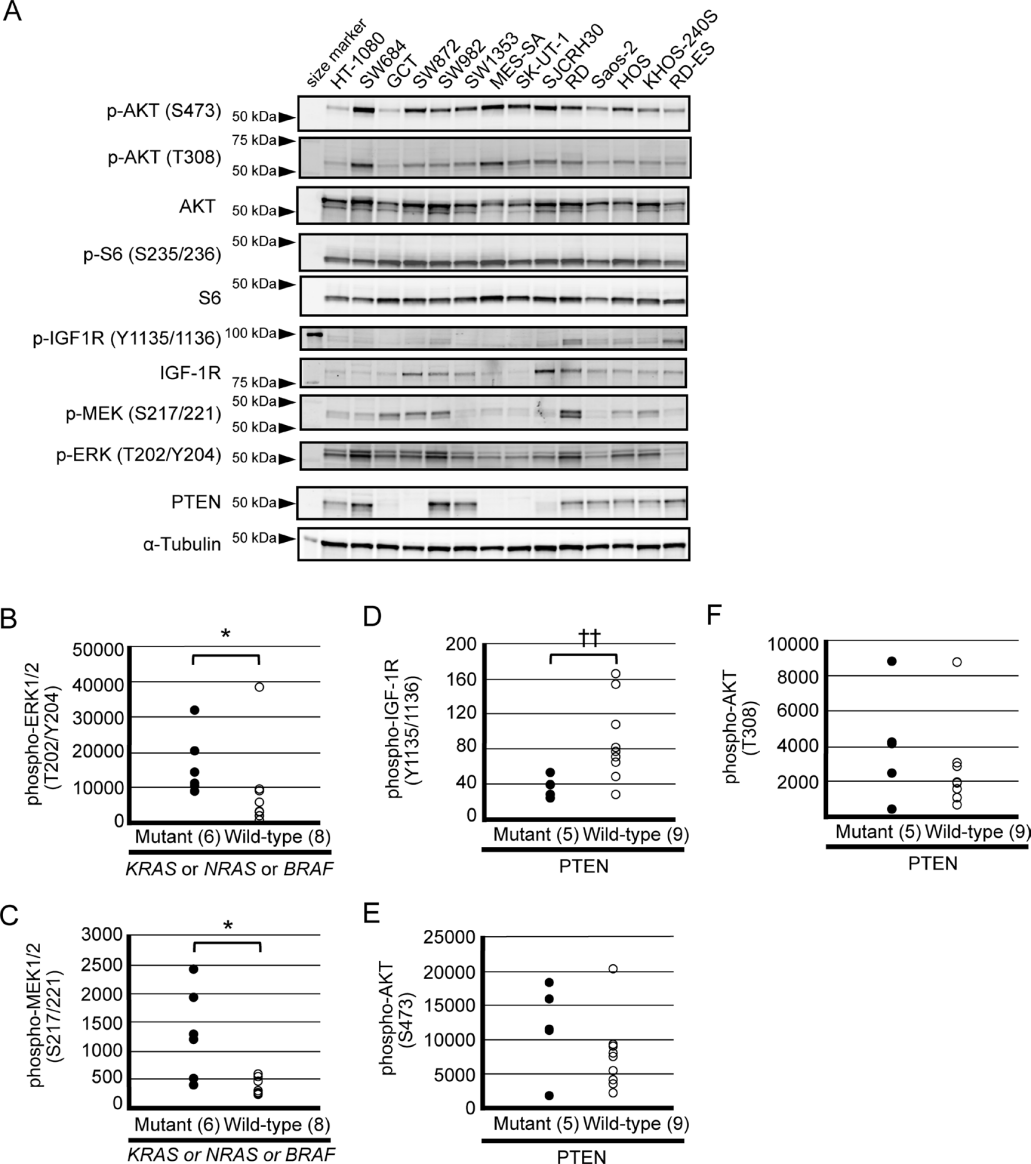
Baseline expression of signaling molecules within the PI3K pathway (AKT, S6, IGF-1R and PTEN) and MEK-ERK pathway in a panel of sarcoma cell lines (**A**) Western blot analysis of phosphorylated AKT (S473), phosphorylated AKT (T308), total AKT, phosphorylated S6 (S235/236), total S6, phosphorylated IGF-1R (Y1135/1136), total IGF-1R, phosphorylated MEK 1/2 (S217/221), phosphorylated ERK 1/2 (T202/Y204), PTEN and α-tubulin was performed using cellular extracts obtained from each cell line as described in the Materials and Methods section. (**B**–**F**) Detected signals were quantified by an Odyssey CLx Imaging System. Association between aberrant gene status and protein expression levels was determined by a Mann–Whitney *U* test (^*^p < 0.05)/ Welch *t* test (^††^*p* < 0.01).

We next examined the expression and the activation status of signaling proteins involved in PI3K-AKT-mTOR and RAS-MEK-ERK pathways by immunoblot analysis, as shown in Figure 1A. Baseline expression of phosphorylated AKT (S473 and T308) and S6 (S235/236) were detected in most of the cell lines examined, suggesting substantial activation of AKT consistent with previous findings [32–36]. Expression of PTEN was significantly detected in nine of the cell lines, whereas it was barely detectable or absent in the remaining five cell lines.

We then investigated the association between gene mutations/expression and phosphorylation levels. Interestingly, cell lines harboring a gain of function mutation in either *NRAS*, *KRAS* or *BRAF* genes expressed phosphorylated MEK and ERK proteins at a significantly higher level than wild-type cell lines (Figure 1B and 1C), whereas no such association was observed regarding phosphorylated AKT nor S6 (data not shown). Unexpectedly, PTEN expression status did not associate with phosphorylated AKT levels; instead, it associated with phosphorylated IGF-1R levels (Figure 1D–1F). Besides those indicated above, no significant associations were found between other point mutations and the expression levels of PI3K/AKT and MEK signaling proteins (data not shown).

### Determination of antiproliferative efficacy patterns of PI3K inhibitors and other molecularly targeted drugs/chemotherapeutic drugs across the sarcoma cell line panel

**Figure 2:**
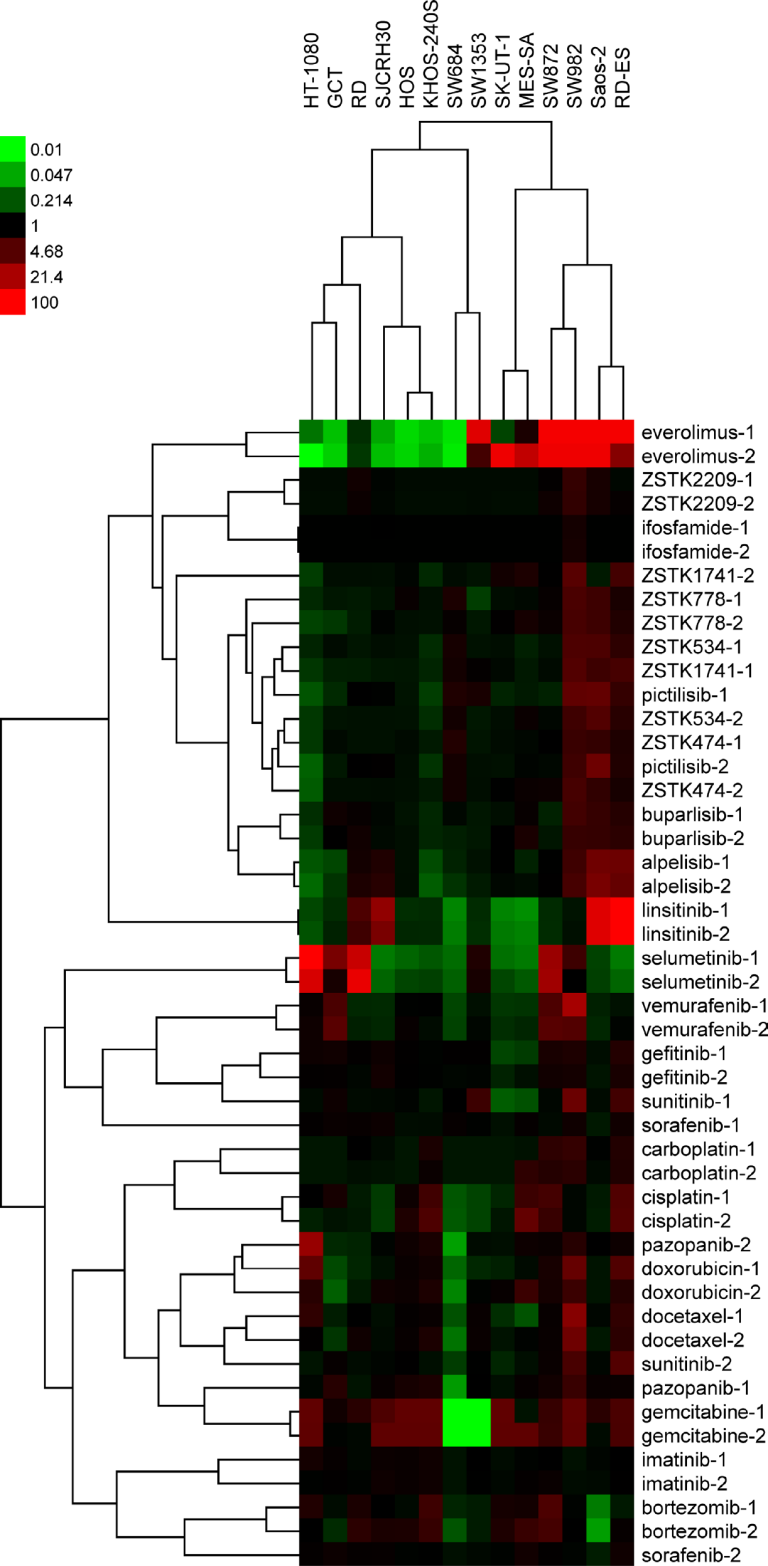
Hierarchical clustering of antiproliferative fingerprints of 24 anticancer compounds across 14 sarcoma cell lines and their relative antitumor activities visualized *via* a heatmap The GI_50_ concentration in each cell line was determined and log-transformed. The compounds were clustered on the basis of their correlations with other compounds (average-linkage clustered with Pearson correlation metric). Cluster analysis and visualization by heatmap was performed by using Cluster3.0/TreeView software (Stanford University). Black represents the average |log GI_50_| for each compound across the 14 cell lines assessed. Red and green represent sensitivity and resistance by 100-fold, respectively.

We next examined the antiproliferative effect of PI3K inhibitors, as well as other molecularly targeted drugs and chemotherapeutic drugs, in each of the cell lines within the sarcoma cell line panel. A total of 24 antitumor agents were tested and are listed in Table 2. Dose-response curves for each drug against all 14 cell lines is presented in Supplementary Figure 1, with the corresponding 50% growth inhibition (GI_50_) concentrations also calculated (Supplementary Table 1). Then, we performed *COMPARE* analysis of the GI_50_ patterns across the 14 cell lines, or “*fingerprints*”, of ZSTK474 with those of other agents. As shown in Supplementary Table 2, the most highly correlated drug was ZSTK474 itself, which was done as an independent experiment. PI3K inhibitors including derivatives of ZSTK474 (ZSTK778, ZSTK534 and ZSTK1741), pictilisib (GDC-0941), buparlisib (BKM120) and alpelisib (BYL-719) were highly ranked, and everolimus, an allosteric inhibitor of mTOR, appeared after the PI3K inhibitors. Based on the correlation coefficients obtained, we performed hierarchical cluster analysis of the 24 drugs assessed (Figure 2). As expected, most of the pairs determined by two independent experiments resembled each other and were tightly clustered. Consistent with *COMPARE* analysis, PI3K inhibitors were sorted into one cluster and their fingerprints were clearly different from other molecularly targeted agents and chemotherapeutics. In fact, ZSTK474 was broadly effective across the 14 cell lines tested and the GI_50_ concentrations were distributed within a 10-fold range (0.1 to 1 μM; Supplementary Figures 1D and 2). In contrast, regarding the fingerprints for other molecularly targeted drugs, such as selumetinib and linsitinib, large variances were observed in respect of observed GI_50_ concentrations across the cell line panel. While these drugs were particularly potent against 3–4 cell lines tested, they minimally affected the remaining cell lines tested. As for other drugs tested, gemcitabine caused strong growth inhibition to most of the sarcoma cell lines; however it was ineffective against SW684 and SW1353 cells. Docetaxel, doxorubicin and bortezomib also exhibited potent growth inhibitory activity to sarcoma cells, with a cytocidal effect observed by doxorubicin and bortezomib in these assays (Supplementary Figure 1A and 1B). The multikinase inhibitors sorafenib and sunitinib showed moderate antitumor effects and exerted cytotoxicity at high concentrations (Supplementary Figure 1B and 1C).

**Table 2:** List of 24 antitumor agents examined by using sarcoma cell line panel

Agents	Mode of action	Manufacturer
Molecularly targeted agents		
ZSTK474	Pan-PI3K inhibitor	Zenyaku Kogyo Co., Ltd.
buparlisib (BKM120)	Pan-PI3K inhibitor	Novartis International AG
pictilisib (GDC-0941)	Pan-PI3K inhibitor	Genentech inc.
alpelisib (BYL719)	PI3K-alpha specific inhibitor	Novartis International AG
ZSTK534	Metabolites of ZSTK474	Zenyaku Kogyo Co., Ltd.
ZSTK778		
ZSTK1741		
ZSTK2209		
linsitinib (OSI906)	IGF-1R inhibitor	OSI Pharmaceuticals, Inc.
everolimus (RAD-001)	mTOR inhibitor	Novartis International AG
selumetinib (AZD6244)	MEK1/2 inhibitor	AstraZeneca plc.
vemurafenib	BRAF (V600E) inhibitor	Genentech inc.
gefitinib	EGFR inhibitor	AstraZeneca plc.
sorafenib	Multikinase inhibitor	Bayer AG & Onyx Pharmaceuticals Inc.
pazopanib	Angiogenesis (multi-kinase inhibitor)	Novartis International AG
imatinib	Bcr-Abl inhibitor	Novartis International AG
sunitinib	Multikinase inhibitor	Pfizer Inc.
bortezomib	Proteasome inhibitor	The Takeda Oncology Company (Millennium Pharmaceuticals, Inc.)
Chemotherapy agents		
doxorubicin	Antineoplastic antibiotics	Kyowa Hakko Kirin Co., Ltd.
docetaxel	Microtubule depolymerization	Sanofi S. A.
gemcitabine	Cytosine analogue	Eli Lilly and Company
cisplatin	DNA cross-linker	Bristol-Myers Squibb Co.
carboplatin	DNA cross-linker	Bristol-Myers Squibb Co.
ifosfamide	DNA alkylator	Baxter International Inc. etc.

Regarding the dendrogram of cell lines, SK-UT-1 and MES-SA, both of which were of leiomyosarcoma origin, and HOS and KHOS-240S, of osteosarcoma origin, were sorted into the same clusters. Other cell lines classified independently regardless of the histological categorization; for example, the fibrosarcoma cell lines HT-1080 and SW684 were sorted into separate clusters (Figure 2).

### Association analysis between drug sensitivity and activation status of signaling pathways

We have thus far studied drug efficacy and the activation status of signaling pathways across the sarcoma cell line panel. Using these data, we first examined the association between fingerprints of drug efficacy and mutation status. The most prominent association identified in this screen was found between efficacy of the MEK inhibitor selumetinib and the gain of function mutation in either *NRAS*, *KRAS* or *BRAF* genes (Supplementary Figure 3). Notably, HT-1080 and RD cells, which both harbor a gain of function mutation on Q61 residue of *NRAS*, were extremely sensitive to selumetinib. As expected, three of the cell lines which harbored a *BRAF* V600E mutation, namely SW982, SW872 and GCT, exhibited sensitivity to the mutant selective BRAF inhibitor, vemurafenib. In contrast, none of the gene mutations examined correlated with sensitivity to PI3K inhibitors (Supplementary Figure 2).

We secondly examined the correlation between drug efficacy and activation status; *i.e.*, phosphorylation levels of signaling proteins. As shown in Supplementary Table 3, none of the phosphorylated proteins, including AKT and ribosomal S6 protein, correlated with PI3K inhibitor efficacy, as examined in this study. Instead, we found significant negative correlations between phosphorylated AKT (T308) and sensitivity to pazopanib (*r* = –0.70), docetaxel (*r* = –0.63) or gemcitabine (*r* = –0.57). In addition, we found a significant positive correlation between phosphorylated IGF1R and the IGF1R-TKI, linsitinib (*r* = 0.63) or the PI3K-alpha specific inhibitor alpelisib (*r* = 0.55), and between phosphorylated MEK and sensitivity to selumetinib (*r* = 0.66) or vemurafenib (*r* = 0.55).

### *In vitro* proof of concept of PI3K inhibition upon treatment with ZSTK474

**Figure 3:**
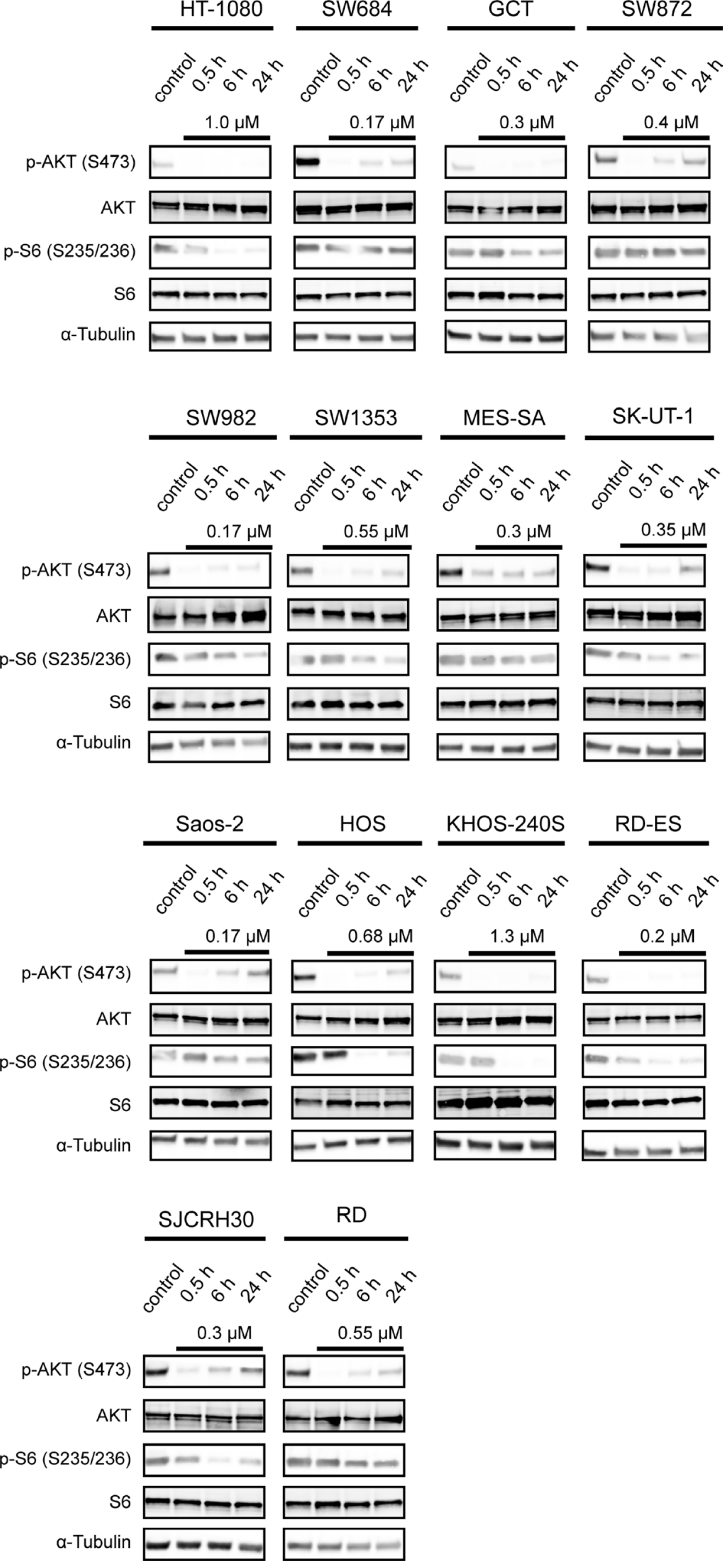
Effect of ZSTK474 on phosphorylation status of AKT in sarcoma cell lines *in vitro* Cells were treated without or with ZSTK474 at the indicated concentrations for 0.5, 6 and 24 h. Samples were lysed and immunoblotted to detect phosphorylated AKT (S473), total AKT, phosphorylated S6 (S235/236), total S6 and α-tubulin as described in the Materials and Methods section.

To confirm proof of concept of PI3K inhibition *in vitro*, we examined the phosphorylation status of PI3K-downstream signaling molecules after treatment with ZSTK474. As shown in Figure 3, the expression levels of phosphorylated AKT (S473) and phosphorylated S6 protein (S235/236) were downregulated upon treatment with ZSTK474 at a concentration required for 50% growth inhibition in each of 14 sarcoma cell lines within 30 min and 6 h, respectively. These results clearly indicated the proof of concept that ZSTK474 certainly inhibited the PI3K-downstream signaling pathway in sarcoma cells, in parallel to growth inhibition.

### *In vivo* antitumor activity of ZSTK474 in sarcoma xenograft models

**Figure 4:**
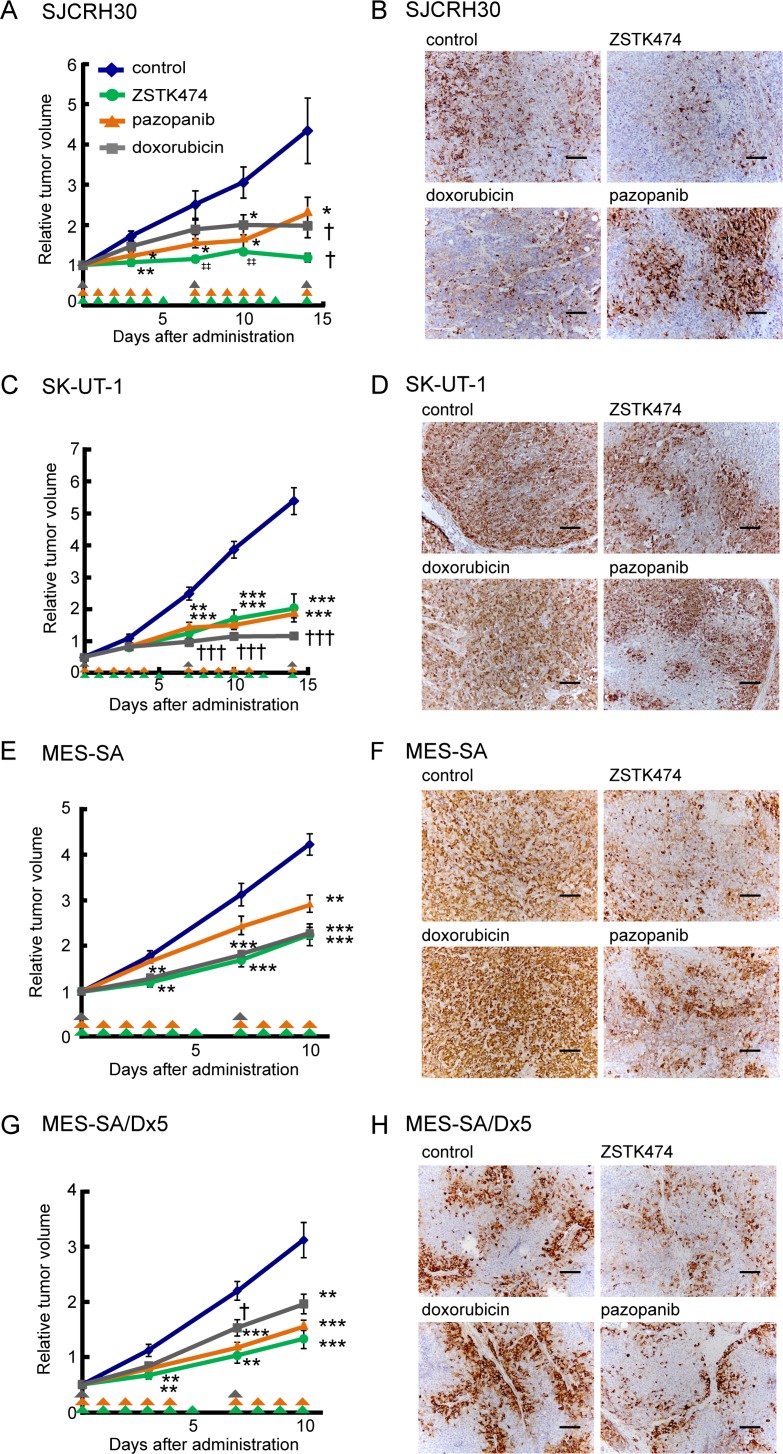
*In vivo* antitumor activity of ZSTK474 against sarcoma cell lines and the multi-drug resistant cell line (MES-SA/Dx5), in comparison to conventional antitumor drugs clinically used for sarcoma (**A**, **C**, **E**, **G**) Antitumor activity of ZSTK474, pazopanib and doxorubicin against SJCRH30 (A), SK-UT-1 (C), MES-SA (E) and MES-SA/Dx5 (G) *in vivo*. Mice were treated with each agent for 15 days (A, C) or 10 days (E, G). ZSTK474 (400 mg/kg of body weight/day) and pazopanib (200 mg/kg of body weight/day) were orally administrated on the days indicated by green and orange arrowheads, respectively. Doxorubicin (10 mg/kg of body weight/week) was intravenously injected on the days indicated by grey arrowheads. The significance of any differences present was determined by either Student's *t* test (^*^p < 0.05; ^**^p < 0.01; ^***^p < 0.001), Welch *t* test (^†^*p* < 0.05; ^†††^*p* < 0.001) or Mann–Whitney *U* test (^‡‡^*p* < 0.01). Symbols were indicated as follows: blue diamonds, control group; green circles, ZSTK474; orange triangles, pazopanib; grey squares, doxorubicin. (**B**, **D**, **F**, **H**) Phosphorylated S6 ribosomal protein (Ser235/236) was detected by immunohistochemistry in control tumor or tumors from animals treated with ZSTK474, pazopanib or doxorubicin in the case of xenografted tumors derived from SJCRH30 (B), SK-UT-1 (D), MES-SA (F) and MES-SA/Dx5 (H) cells. Tumors were resected on day 15 (SJCRH30), 16 (SK-UT-1) or 10 (MES-SA and MES-SA/Dx5). Scale bars: 100 μm.

Given that PI3K inhibitors showed antiproliferative activity in sarcoma cell lines *in vitro*, we next exploited sarcoma xenograft models to determine antitumor activity of ZSTK474 *in vivo*. Of the 14 sarcoma cell lines, three cell lines (SJCHR30, SK-UT-1 and MES-SA) were subcutaneously transplantable and exhibited reproducible tumor growth in nude mice (data not shown). Therefore, we examined the effect of ZSTK474 on growth of xenografted tumors derived from these cell lines, and compared it with that affected by doxorubicin and pazopanib. As shown in Figure 4, ZSTK474 exerted comparable antitumor effects (treated/control ratio [T/C]= 27.4% in SJCRH30, 38.0% in SK-UT-1, 53.0% in MES-SA and 47.0% in MES-SA/Dx5) to both doxorubicin (T/C = 45.6% in SJCRH30, 21.3% in SK-UT-1, 53.8% in MES-SA and 69.4% in MES-SA/Dx5), and pazopanib (T/C = 53.9% in SJCRH30, 35.5% in SK-UT-1, 69.2% in MES-SA and 55.7% in MES-SA/Dx5); notably, ZSTK474 completely inhibited tumor growth of SJCRH30 xenografts. Interestingly, ZSTK474 exerted a similar antitumor effect to both MES-SA and its doxorubicin-resistant variant, MES-SA/Dx5. Immunohistochemistry (IHC) revealed that ZSTK474 efficiently suppressed expression of phosphorylated S6 protein, a PI3K-downstream signaling molecule, whereas neither doxorubicin nor pazopanib suppressed phosphorylated S6 protein. The results provided the proof of concept that ZSTK474 certainly exerted antitumor effects *via* inhibiting the PI3K signaling pathway *in vivo*, as well as *in vitro*.

### Induction of apoptosis and antitumor effects of ZSTK474 against Ewing's sarcoma cells

**Figure 5:**
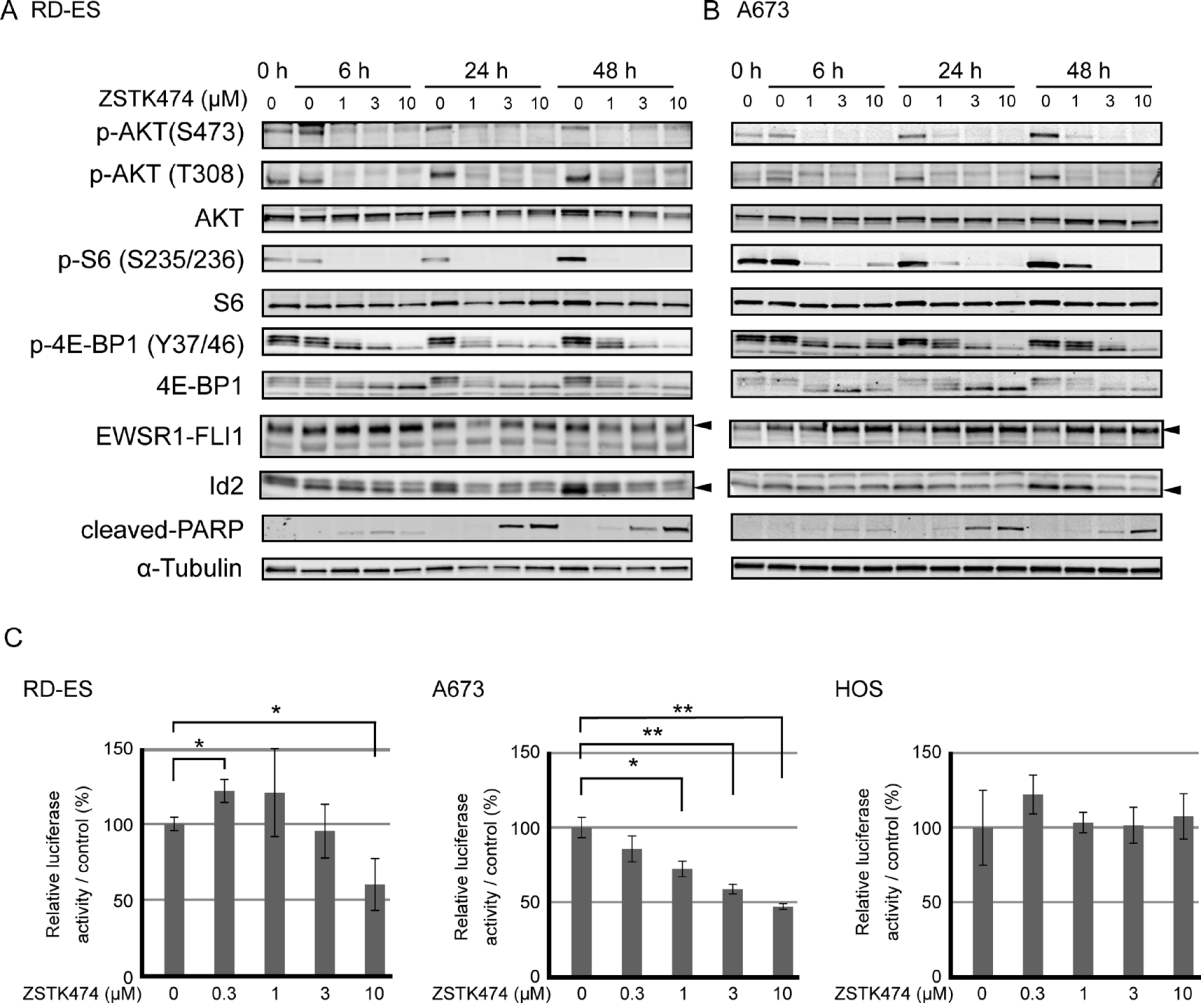
Effect on PI3K-downstream signaling pathway, apoptosis progression, transcriptional activity of EWSR1-FLI1 and protein expression of Id2 upon treatment with ZSTK474 in Ewing's sarcoma cell lines (**A**, **B**) RD-ES cells (A) and A673 cells (B) were treated with ZSTK474 at the specified concentrations for the indicated time. Western blot analysis of phosphorylated AKT (S473), phosphorylated AKT (T308), total AKT, phosphorylated S6 (S235/236), total S6, phosphorylated 4E-BP1 (T37/46), total 4E-BP1, EWSR1-FLI1, Id2, cleaved-PARP and α-tubulin were performed as described in the Materials and Methods section. (**C**) The effect of ZSTK474 on transcriptional activity from an Id2 promoter in Ewing's sarcoma cell lines as determined by reporter assays. The Ewing's sarcoma cell lines, RD-ES and A673, and an osteosarcoma cell line, HOS, were co-transfected with the reporter plasmids pGL3-Id2-2755 (firefly luciferase) and pGL3-CMV (Renilla luciferase). Cells were treated with ZSTK474 at the indicated concentrations for 24 h. Relative activities of firefly luciferase (derived from pGL3-Id2-2755) to Renilla luciferase (from pGL3-CMV) were calculated and normalized to drug-untreated control. Experiments were performed in triplicate. The significance of any differences present was determined by a Student's *t* test (^*^p < 0.05; ^**^p < 0.01).

We previously reported that the antitumor effect of ZSTK474 in carcinoma cells is mediated by a cytostatic effect *via* G1 arrest of the cell cycle, but not by a cytotoxic effect *via* apoptosis, using carcinoma cells from various origins both *in vivo* and *in vitro* [28–30]. In the present study, to investigate the involvement of apoptosis in the antitumor effects of ZSTK474 in sarcoma cells, 14 sarcoma cell lines were treated with ZSTK474 at a higher concentration than GI_50_ for an extended period (48 h, Supplementary Figure 4). Unexpectedly, emergence of cleaved poly (ADP-ribose) polymerase (PARP), one of the hallmarks of apoptosis, was observed selectively in RD-ES, an Ewing's sarcoma cell line, and SJCRH30, an alveolar rhabdomyosarcoma cell line, whereas no such event was observed in other sarcoma cell lines, except that a slight increase was observed in the Saos-2, osteosarcoma cell line. These results suggested that induction of apoptosis could be involved in the selective antitumor effect of ZSTK474 in certain types of sarcoma cells. To characterize cellular responses to ZSTK474 in Ewing's sarcoma cells in more detail, we utilized A673 and RD-ES cells to perform time course and dose-response experiments (Figure 5A, 5B). Dephosphorylation of PI3K-downstream signaling proteins, including AKT (on S473 and T308 residues), ribosomal S6 protein (on S235/236 residues) and 4E-BP1 (on T37/46 residues) were observed within 6 h following ZSTK474 treatment, with effects lasting at least 48 h after drug exposure. Interestingly, cleaved PARP was detected within 6 h after exposure of ZSTK474 at a concentration of 1 μM or more, suggesting that progression of apoptosis started earlier than 6 h. As expected, apoptotic cells increased in a dose-dependent manner in both Ewing's sarcoma cell lines following ZSTK474 treatment. From these results, we concluded that Ewing's sarcoma cells underwent apoptosis upon treatment with ZSTK474 *in vitro*.

### Effect of ZSTK474 on EWSR1-FLI1 and its transcriptional activity

The most common chromosomal translocation found in Ewing's sarcoma, t(11;22)(q24;q12), generates an aberrant transcription factor *EWSR1-FLI1* fusion gene. Both the RD-ES and A673 cell lines expressed the EWSR1-FLI1 fusion protein. Previous reports have shown that protein expression of EWSR1-FLI1 fusion was attenuated upon treatment with the PI3K/mTOR dual inhibitor dactolisib (BEZ235) [37], suggesting the potential involvement of EWSR1-FLI1 in the antitumor effect of ZSTK474. However, protein expression of EWSR1-FLI1 did not attenuate upon treatment with ZSTK474 in either the RD-ES or A673 cell lines (Figure 5A, 5B). Instead, we found that ZSTK474 reduced the expression level of Id2, which was transcriptionally regulated by EWSR1-FLI1, in the Ewing's sarcoma cell lines.

Then, we next examined the effect of ZSTK474 on transcriptional activity of EWSR1-FLI1 by reporter assays. As shown in Figure 5C, the transcriptional activity from the Id2 promoter was suppressed upon treatment with ZSTK474 in a dose-dependent manner. In contrast, no such attenuation of reporter activity was observed upon ZSTK474 treatment in the *EWSR1-FLI1*-negative osteosarcoma cell line, HOS. This result suggested that downregulation of Id2 protein expression observed in Ewing's sarcoma cell lines upon treatment with ZSTK474 could be mediated *via* transcriptional inactivation of EWSR1-FLI1 from the Id2 promoter.

### *In vivo* antitumor activity of ZSTK474 against Ewing's sarcoma xenografts

**Figure 6:**
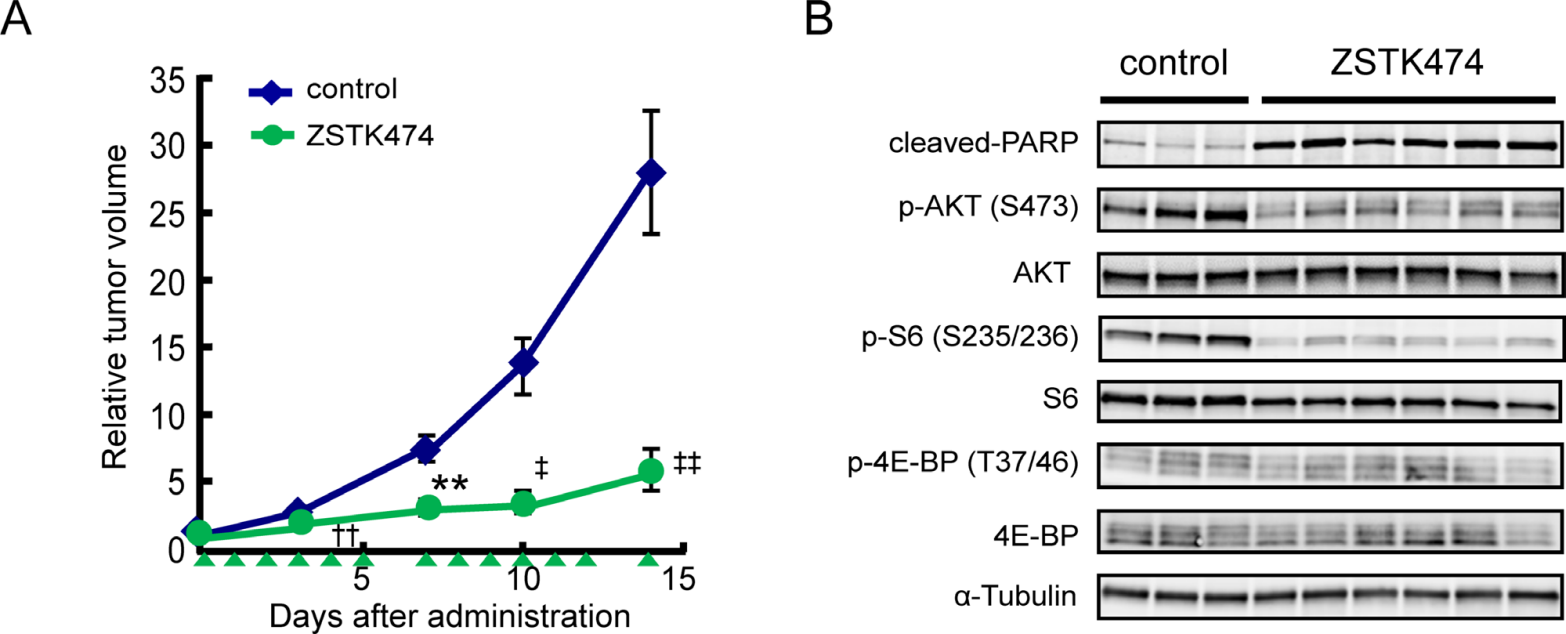
*In vivo* antitumor activity of ZSTK474 in Ewing's sarcoma cell lines (**A**) Antitumor activity of ZSTK474 against A673 xenografts *in vivo*. Tumor-bearing mice were administered with ZSTK474 (400 mg/kg) on the days indicated by green arrowheads. Tumor volumes of each mice was measured and the mean (±S.E.) relative tumor volume of each group was calculated. The significance of the differences present was determined by either a Student's *t* test (^**^p < 0.01), Welch *t* test (^††^*p* < 0.01) or Mann–Whitney *U* test (^‡^*p* < 0.05; ^‡‡^*p* < 0.01). Symbols were indicated as follows: blue diamonds, control group; green circles, ZSTK474. (**B**) Western blot analysis of cleaved-PARP, phosphorylated AKT (S473), total AKT, phosphorylated S6 (S235/236), total S6, phosphorylated 4E-BP1 (T37/46), total 4E-BP1 and α-tubulin in A673 xenografted tumor samples after administration with ZSTK474. Emergence of cleaved-PARP indicated the progression of apoptosis.

We next examined the antitumor activity of ZSTK474 against Ewing's sarcoma cells *in vivo*. A673 cells were subcutaneously transplanted in nude mice, with the rapid growth of tumors subsequently observed. ZSTK474 administration for two weeks exerted a robust antitumor activity against A673-derived tumors (T/C = 20.8%, Figure 6A). Immunoblot analysis of tumor samples resected from nude mice revealed that administration of ZSTK474 for two weeks downregulated the PI3K-downstream signaling pathway, as determined by dephosphorylation of AKT and ribosomal S6 protein (Figure 6B). Moreover, cleaved PARP was selectively detected from the samples that had been derived from ZSTK474 administrated-animals, suggesting that A673 cells underwent apoptosis upon ZSTK474 administration *in vivo*.

### Induction of apoptosis and antitumor effect of ZSTK474 on synovial sarcomas

**Figure 7:**
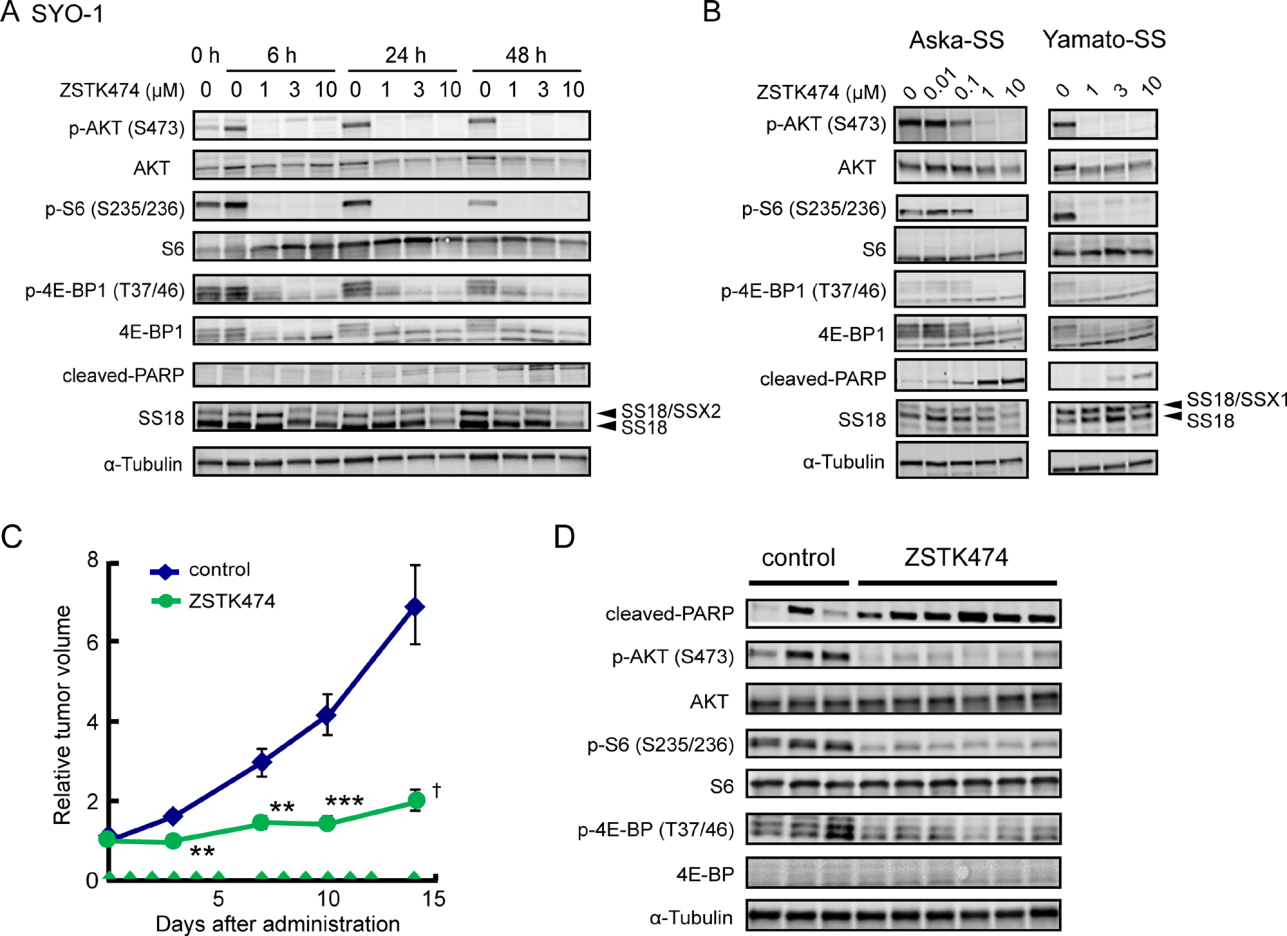
Effect of ZSTK474 on PI3K-downstream signaling pathway, apoptosis progression and *in vivo* antitumor activity in synovial sarcoma cell lines (**A**, **B**) Synovial sarcoma cell lines, SYO-1 (A), Aska-SS and Yamato-SS (B) were treated with ZSTK474 at the indicated concentrations for the indicated time (A) or 48 h (B). Expression of phosphorylated AKT (Ser473), total AKT, phosphorylated S6 (Ser235/236), total S6, phosphorylated 4E-BP1 (Thr37/46), total 4E-BP1, cleaved-PARP, SS18 and α-tubulin was examined by Western blot analysis as described in the Materials and Methods section. (**C**) Antitumor activity of ZSTK474 against SYO-1 xenografts *in vivo*. Tumor-bearing mice were administered with ZSTK474 (400 mg/kg) on the days indicated by green arrowheads. Tumor volume of each mice was measured and the mean (±S.E.) relative tumor volume of each group were calculated. The significance of any differences present was determined by a Student's *t* test (^**^p < 0.01; ^***^p < 0.001) or Welch *t* test (^†^*p* < 0.05). Symbols were indicated as follows: blue diamonds, control group; green circles, ZSTK474. (**D**) Western blot analysis of cleaved-PARP, phosphorylated AKT (Ser473), total AKT, phosphorylated S6 (S235/236), total S6, phosphorylated 4E-BP1 (T37/46), total 4E-BP1 and α-tubulin in SYO-1 xenografted tumor samples after administration with ZSTK474.

Induction of apoptosis in Ewing's sarcoma cell lines prompted us to examine another chromosomal translocation-positive sarcoma subtype. SYO-1, a synovial sarcoma cell line carrying *SS18* (*SYT*)-*SSX2* fusion gene, underwent apoptosis upon treatment with ZSTK474, as determined by emergence of cleaved PARP (Figure 7A). Similar results were obtained in two additional synovial sarcoma cell lines, Aska-SS and Yamato-SS (Figure 7B) [38]. We then examined the effect of ZSTK474 on SS18-SSX chimera protein expressed in synovial sarcoma cell lines. In SYO-1 and Aska-SS cells, protein expression of SS18-SSX, as well as SS18, was reduced upon treatment with ZSTK474, while no significant reduction was observed in Yamato-SS cells.

We finally examined the *in vivo* antitumor effect of ZSTK474 against synovial sarcoma xenografts. As expected, ZSTK474 suppressed the growth of SYO-1 tumors (T/C = 29.2%, Figure 7C). Immunoblot analysis of tumor samples revealed that ZSTK474 certainly downregulated the PI3K-downstream signaling pathway and induced apoptosis, as determined by detection of cleaved PARP (Figure 7D). These results suggested that ZSTK474 induced apoptosis and exerted significant antitumor efficacy against synovial sarcoma, as well as Ewing's sarcoma.

### Anti-angiogenic effect of ZSTK474 *in vivo* sarcoma xenograft model

**Figure 8:**
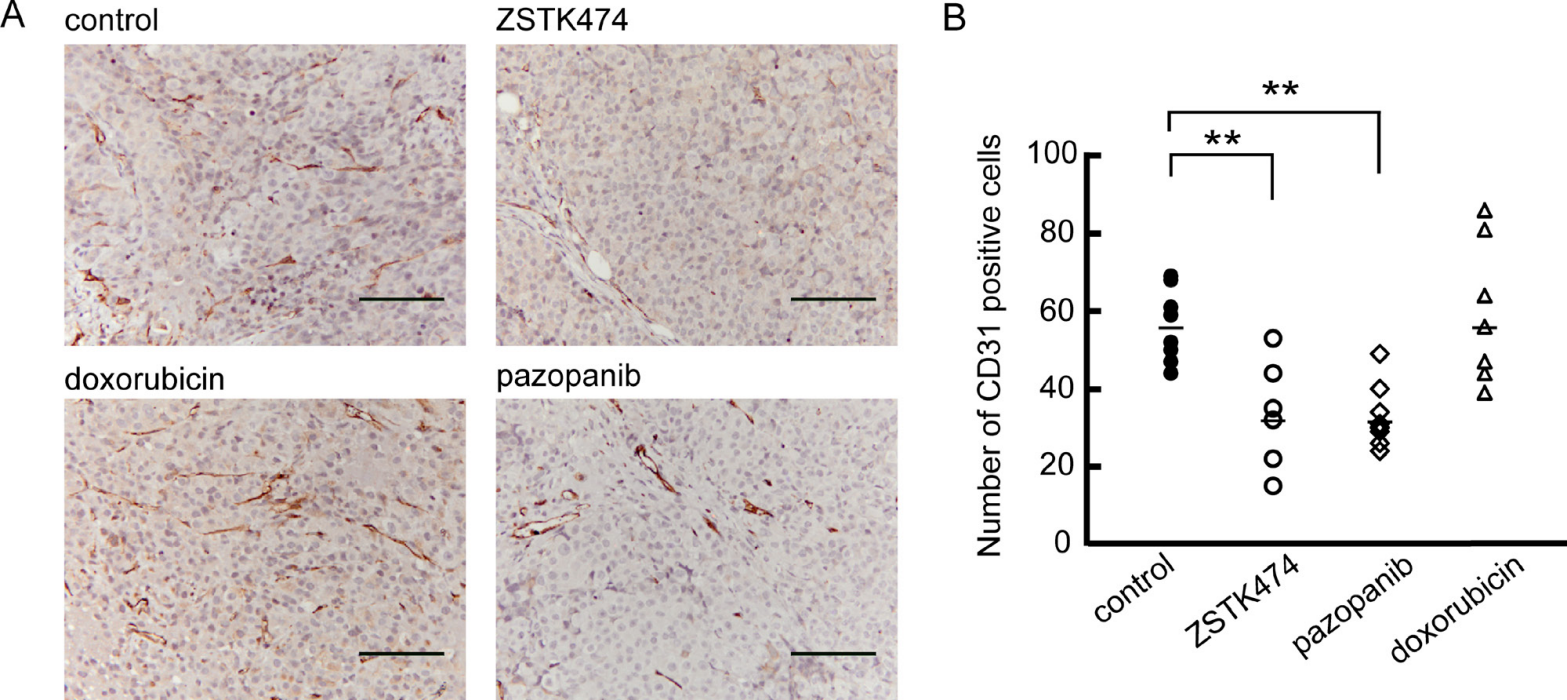
Anti-angiogenic activity of ZSTK474 against MES-SA xenograft *in vivo* (**A**) Representative images of tumor vascularization in an MES-SA tumor tissue section resected from control mice or mice administered with ZSTK474, pazopanib and doxorubicin, respectively. Vascular endothelial cells were detected by immunohistochemistry using an anti-CD31 antibody. Scale bars: 100 μm. (**B**) The number of CD31-positive cells in the MES-SA tumor tissue was counted at 7 or 8 different separate positions and the average calculated. ZSTK474 effectively repressed the number of CD31 positive cells. The significance of any differences present was determined by a Student's *t* test (^**^p < 0.01).

We previously reported that ZSTK474 exerted an antiangiogenic effect in respect of a carcinoma xenograft model *in vivo* [39]. We, therefore, examined the anti-angiogenic effect of ZSTK474 on sarcoma xenografts by IHC analysis (Figure 8A). In the control groups, formation of CD31-positive tumor vessels was observed in the MES-SA xenograft, as well as those derived from SJCRH-30, SK-UT-1, MES-SA/Dx5 and A673 cells (Supplementary Figure 5A). However, blood vessel density was significantly reduced in the tumors resected from mice after consecutive administration of ZSTK474 for two weeks. Reduction of tube formation was also observed in the SYO-1 xenograft resected from mice after ZSTK474 administration (Supplementary Figure 5B). Similar results were obtained after administration of an angiogenesis inhibitor, pazopanib; however, blood vessels remained unchanged after administration of doxorubicin. These results suggested that ZSTK474 efficiently inhibited angiogenesis in sarcoma xenografts *in vivo* to a similar extent to pazopanib.

## DISCUSSION

In the present study, we characterized the antitumor profile of PI3K inhibitors, in particular ZSTK474 which we developed, against sarcomas in preclinical models by exploiting a panel of sarcoma cell lines derived from various origin; in this respect, effects on tumor growth, PI3K-downstream signaling pathway and apoptosis induction *in vitro* and *in vivo* were assessed. *COMPARE* analysis and hierarchical clustering of fingerprints of tumor growth inhibition revealed that ZSTK474 highly resembled its closely related derivatives and other PI3K inhibitors in terms of effects, which was also clearly different from other molecularly-targeted agents and chemotherapeutics. The results strongly supported our hypothesis that these compounds shared a specific mode of action, *i.e.*, similarity of their fingerprints was as a result of PI3K inhibition against sarcoma cells. In fact, ZSTK474 effectively suppressed tumor cell growth at submicromolar concentrations, with the variance of GI_50_ concentrations across the 14 cell lines being small (within a 10-fold range) compared to the MEK inhibitor selumetinib (~1000-fold range). These results suggested that ZSTK474 effectively suppressed tumor growth in sarcoma cell lines irrespective of their subtype. Moreover, ZSTK474 downregulated phosphorylation of PI3K-downstream signaling factors including AKT and ribosomal S6 protein in all of the sarcoma cell line tested, suggesting proof of concept of PI3K inhibition.

In our previous study using the JFCR39 carcinoma cell line panel, gain of function mutations of the *KRAS* or *BRAF* genes and overexpression of IGF1R were found to be negative predictors whereas high expression of phosphorylated AKT was observed to be a positive predictor for ZSTK474 efficacy [40]; however, neither gain of function mutations of *KRAS*/*NRAS*/*BRAF* nor overexpression of IGFR/phosphorylated AKT displayed any significant correlation with ZSTK474 efficacy in the sarcoma panel assessed here. HT-1080, one of the less effective cell lines examined, harbored gain of function mutation of *NRAS*, suggesting that *NRAS* mutation could be involved ZSTK474 inefficacy; however, RD, another *NRAS* mutant cell line, responded to ZSTK474 at a moderate level. Further studies are needed to clarify whether *NRAS* mutation predicts ZSTK474 inefficacy. In addition, we could not determine whether *PIK3CA* mutation could predict the efficacy of ZSTK474, since none of the 14 sarcoma cell lines examined harbored a gain of function mutation of *PIK3CA* despite gain of function mutations in this genes being commonly found in in 18–25% of patients with liposarcomas [41, 42]. Loss of PTEN expression seemed to have no association with ZSTK474 efficacy.

In contrast to growth inhibitory activity, apoptosis induction was selectively observed following ZSTK474 treatment. As summarized in Table 3, extensive induction of apoptosis was observed only in two of the 14 cell lines across the sarcoma panel, both of which were derived from chromosomal translocation-positive sarcomas (*EWSR1-FLI1* in Ewing's sarcoma RD-ES cell line and *PAX3-FOXO1* in alveolar rhabdomyosarcoma SJCRH30 cell line, respectively) [43, 44]. We additionally examined the effect of ZSTK474 in another Ewing's sarcoma cell line, A673, and found that it also induced apoptosis in this context. Interestingly, we also examined the SYO-1 cell line, which is derived from another chromosomal translocation-positive subtype, synovial sarcoma harboring *SS18-SSX2* fusion gene [45], and found that it underwent apoptosis following ZSTK474 treatment. The rest of the 12 sarcoma cell lines used in this study did not undergo apoptosis following ZSTK474 treatment, except that a slight increase was observed in the Saos-2 osteosarcoma cell line. This cell line has not been reported to express an oncogenic fusion gene generated by specific chromosomal translocation, corroborating our hypothesis that ZSTK474 could preferentially induce apoptosis in chromosomal translocation-positive sarcomas. Nevertheless, one cannot exclude the possibility of an, as yet, unknown chromosomal translocation event present in these cell lines that may confound the results.

**Table 3:** Summary of *in vitro* and *in vivo* antitumor activities of ZSTK474 across the sarcoma cell lines

Subtype		Cell line	Fusion gene	Panel	*in vitro* activities		*in vivo* activities		
					GI_50_ (μM)	Apoptosis induction	T/C (%)	Apoptosis induction	Anti-angiogenic effect
Fibrosarcoma		HT-1080	-	●	0.94	-		n.d.	
		SW684	-	●	0.18	-		n.d.	
Giant cell sarcoma		GCT	-	●	0.41	-		n.d.	
Leiomyosarcoma		SK-UT-1	-	●	0.42	-	38.0	-	●
Rhabdomyosarcoma	alveolar	SJCRH30	*PAX3-FOXO1*	●	0.46	●	27.4	●	●
	embryonic	RD	-	●	0.47	-		n.d.	
Osteosarcoma		HOS	-	●	0.41	-		n.d.	
		KHOS-240S	-	●	0.55	-		n.d.	
		Saos-2	-	●	0.14	▲		n.d.	
Liposarcoma		SW872	-	●	0.35	-		n.d.	
Synovial sarcoma		SW982	-	●	0.12	-		n.d.	
		SYO-1	*SS18-SSX2*	-	0.031	●	29.2	●	●
		Yamato-SS	*SS18-SSX1*	-	0.023	●		n.d.	
		Aska-SS	*SS18-SSX1*	-	0.0039	●		n.d.	
Chondrosarcoma		SW1353	-	●	0.50	-		n.d.	
Ewing's sarcoma		RD-ES	*EWSR1-FLI1*	●	0.20	●		n.d.	
		A673	*EWSR1-FLI1*	-	0.32	●	20.8	●	●
Uterine sarcoma		MES-SA	-	●	0.40	-	53.0	-	●
		MES-SA/Dx5	*-*	*-*	0.31	-	47.0	n.d.	●

●: yes; ▲: marginal; -: no; n.d.: not determined.

Here, we demonstrated that ZSTK474 partially downregulated transcriptional activity of the EWSR1-FLI1 transcription factor from the Id2 promoter *via* a reporter assay, and suppressed expression of the Id2 protein in both of the Ewing's sarcoma cell lines examined. Actually, a previous study demonstrated that the EWSR1-FLI1 fusion could transform cells [46], and downregulation of EWSR1-FLI1 by RNA interference could induce growth arrest in Ewing's sarcoma [47]. The precise mechanisms by which ZSTK474 downregulated EWSR1-FLI1, and by which mechanism their downregulation rendered cells susceptible to ZSTK474-induced apoptosis remains to be elucidated. Similarly, it has been reported that *SS18*-*SSX* fusion genes are capable of transforming cells and knockdown of SS18-SSX expression can greatly reduce viability of synovial sarcoma cells, as determined by colony formation assays [48]. Interestingly, downregulation of SS18-SSX was observed in SYO-1 and Aska-SS cells, but not in Yamato-SS cells upon treatment with ZSTK474 (Figure 7B). Therefore, further studies are needed to clarify the involvement of SS18-SSX downregulation in progression of apoptosis. It was reported that the synovial sarcoma Aska-SS cell line expressed an activated ALK variant and showed sensitivity to ALK inhibitors, while another synovial sarcoma cell line Yamato-SS overexpressed MET protein and exhibited sensitivity to the ALK/MET dual inhibitor, crizotinib [49, 50]. Since PI3K was shown to be regulated by both ALK and MET signals in some tumor cells [51, 52], selective induction of apoptosis in synovial sarcoma cells was mediated by abrogating these aberrant tyrosine kinase signals. We also examined the expression of PAX3-FOXO1 after ZSTK474 treatment in SJCRH30 (Supplementary Figure 6). ZSTK474 did not suppress PAX3-FOXO1 expression, but electrophoretic mobility shift of PAX3-FOXO1 was observed. PAX3-FOXO1 was shown to be phosphorylated at Ser256 by AKT [53], and thus it was probably due to dephosphorylation of PAX3-FOXO1 *via* PI3K/AKT inhibition. However, the causality between these events observed in chromosomal translocation positive sarcoma cells and the induction of apoptosis were unclear in the context of this study. In fact, we detected neither common nor specific changes in the expression levels of Bcl-2 and IAP (inhibitor of apoptosis) family proteins in these cells upon treatment with ZSTK474 (data not shown). Further studies are needed to understand the precise mechanism of apoptosis induction in such types of sarcoma cells.

In this study, we examined the *in vivo* antitumor effect of ZSTK474 using various sarcoma cell lines subcutaneously xenografted in nude mice. ZSTK474 exerted a significant *in vivo* antitumor effect against all of sarcoma cell lines examined, comparable to the existing antitumor drugs doxorubicin and pazopanib. Of note, *in vivo* effect was most obvious in the three chromosomal translocation-positive sarcoma cell lines that were characterized with apoptosis induction (A673, SJCRH30 and SYO-1; Supplementary Figure 7). In addition, comparable antitumor effects were obtained with a doxorubicin-resistant MES-SA variant MES-SA/Dx5 overexpressing P-glycoprotein compared to its parental cell line, indicating that ZSTK474 could be used for patients experiencing resistance to doxorubicin treatment. Moreover, we clearly demonstrated that ZSTK474 suppressed the number of CD31-positive cells and inhibited blood vessel formation within the tumors, suggestive of an anti-angiogenic effect, in agreement with our previous finding using carcinoma cells [39]. Therefore, anti-angiogenic effect of ZSTK474 could be involved in its potent antitumor activity on sarcoma.

In general, molecularly targeted drugs predominantly exert cytostatic effects on tumor cells [54]. In fact, our previous studies revealed that ZSTK474 induce G1 arrest of cell cycle but hardly induce apoptosis in various carcinoma cell lines both *in vitro* and *in vivo* [28, 30]. In contrast, chromosomal translocation-positive sarcomas underwent apoptosis upon administration of ZSTK474 *in vivo* as well as *in vitro*, as demonstrated in the present study. ZSTK474 has not yet administered to patients with such types of sarcoma; but the present results suggest that ZSTK474 would exert potential clinical benefits on them *via* tumor shrinkage in addition to inhibition of tumor proliferation and warrant further clinical development [55].

## MATERIALS AND METHODS

### Drugs

Buparlisib (BKM-120) and everolimus (RAD-001) were purchased from AdooQ BioScience (Irvine, CA, USA). Sorafenib, doxorubicin and gemcitabine were purchased from Cayman Chemical Company (Ann Arbor, MI). Cisplatin, pictilisib (GDC-0941) and pazopanib were purchased from Cellagen Technology (San Diego, CA, USA). Docetaxel and selumetinib (AZD6244) were purchased from Focus Biomolecules (Plymouth Meeting, PA, USA). Carboplatin and ifosfamide were purchased from LKT Laboratories, Inc. (St. Paul, MN, USA). Alpelisib (BYL-719), vemurafenib, imatinib, sunitinib and gefitinib were purchased from Active Biochemicals Co., Ltd. (Hong Kong, China), Axon Medchem BV. (Groningen, Netherlands), BioVision Inc. (Milpitas, CA, USA), R&D Systems, Inc. (Minneapolis, MN, USA) and Synkinase Pty Ltd. (Parkville, VIC, Australia), respectively. ZSTK474, ZSTK534, ZSTK778, ZSTK1741 and ZSTK2209 were kindly gifted from Zenyaku Kogyo Co., Ltd. For *in vivo* studies, a solid dispersion form of ZSTK474 was suspended with distilled water (Otsuka Pharmaceutical Factory, Inc., Tokushima, Japan). Doxorubicin was purchased from Cayman Chemical Company (Ann Arbor, MI) and dissolved with saline (Otsuka Pharmaceutical Factory, Inc., Tokushima, Japan). Pazopanib was purchased from Cellagen Technology (San Diego, CA), dissolved with DMSO for *in vitro* studies, and suspended with 5% hydroxypropylmethyl cellulose (HPMC) for *in vivo* studies.

### Cell lines and cell culture

Cell lines used in the sarcoma cell line panel assay are listed in Table 1. These cell lines were purchased from the American Type Culture Collection (Manassas, VA), and were maintained in RPMI 1640 (Wako Pure Chemical Industries Ltd., Osaka, Japan) supplemented with 5% (v/v) fetal bovine serum (Nichirei Biosciences, Tokyo, Japan) and 1 μg/ mL kanamycin at 37° C in humid air containing 5% CO_2_. A673 was also purchased from American Type Culture Collection (Manassas, VA) and cultured in the same condition mentioned above. SYO-1 cell line provided by Dr. Akira Kawai (National Cancer Center Hospital, Tokyo, Japan) and Aska-SS and Yamato-SS cell lines provided by Dr. Norifumi Naka (Osaka International Cancer Center, Osaka, Japan) were cultured in the same condition.

### Ion torrent amplicon sequencing

Genomic DNA was isolated from each cell line by DNeasy Blood & Tissue Kit according to the manufacturer's protocols (QIAGEN, Hilden, Germany). Sequencing library construction and Ion torrent amplicon sequencing were performed *via* the TaKaRa Amplicon sequencing service by using the Ion Ampliseq Cancer Hotspot Panel v2, which includes primer sets to amplify 207 amplicons covering approximately 2,800 COSMIC mutations from 50 oncogenes and tumor suppressor genes using human hg19 reference genome as described in the manufacturer's web site (https://www.thermofisher.com/order/catalog/product/4475346).

### Determination of drug efficacy *in vitro*

Inhibitory effects of each agent against the sarcoma cell lines was assessed by using the sulforhodamine B (SRB) assay [56]. Percentage of growth was calculated using the following formula:

Percentage growth (%) = 100 × (T–I)/(C–I)

T: absorbance for the test well after incubation with drug for 48 h

I: absorbance for the test and control well at starting point (addition of drugs),

C: absorbance for the control well (C) after incubation with vehicle for 48 h

The drug concentration of the GI_50_ was calculated as described previously (15, 16). All drugs were tested in duplicate. The GI_50_ values were log transformed and were used for all analyses. Cluster analysis and visualization of heatmaps was performed using the Cluster 3.0 and TreeView software (Stanford University, CA, USA).

### Apoptosis detection

Apoptosis was detected by Hoechst 33342 staining. Briefly, after incubation with ZSTK474 for 48 h, the cells were trypsinized, collected and fixed in ice cold 1% glutarardehyde-PBS (–) at 4° C for 24 h. After washing with PBS (–), the fixed cells were stained with 167 μM Hoechst 33342 (Sigma-Aldrich, MO, USA), and observed *via* fluorescence microscopy. The number of the cells with nuclear condensation and DNA fragmentation was counted and the percentage of apoptotic cells relative to total cells was calculated. At least 100 cells were counted in each group.

### Animals

Female nude mice with BALB/c genetic backgrounds (CAnN.Cg-Foxn1nu/CrlCrlj, 5 weeks old) were purchased from Charles River Japan, Inc. (Kanagawa, Japan), maintained under specific pathogen-free conditions in a 12:12 h light-dark cycle, and provided with sterile food and water *ad libitium*. Animal studies were performed in accordance with the procedures approved by the animal use and care committee of the Japanese Foundation for Cancer Research.

### Determination of drug efficacy *in vivo*

Ectopic sarcoma xenograft models were previously established elsewhere by subcutaneous injection of SJCRH30 [57], SK-UT-1 [58], MES-SA [58], MES-SA/Dx5 [59], A673 [60] or SYO-1 [61] cells. Cells were resuspended in Hank's Balanced Salt Solution (Thermo Fisher Scientific, Waltham, MA) and subcutaneously transplanted into the back of nude mice. Animals were randomly assigned into test groups (each group containing 6 mice) and administration of drugs was performed on the day as indicated in each experiment. ZSTK474 was orally administrated daily at 400 mg/kg of body weight (BW) except for days 6 and 13 from the day of first administration (day 0). Pazopanib was orally administrated daily at 200 mg/kg of BW except for days 5, 6, 12 and 13. Doxorubicin was intravenously injected weekly at 10 mg/kg of BW. The control group mice were administrated with 5% HPMC. Antitumor effects were evaluated for 14 days in SJCRH30-, 14 days in SK-UT-1-, 10 days MES-SA-, 10 days in MES-SA/Dx5-, 14 days in A673-, 14 days in SYO-1- xenografted mice. Tumor volume was measured by calipers twice per week, and the tumor weight (TW) was calculated using the following formula:

TW = (L × W^2^)/2,

L: the longest dimension of the tumor

W: the width dimension of the tumor

The antitumor efficacy was evaluated by T/C, which was calculated using the following formula:

Treated/control ratio [T/C] (%) = 100 × average TW of drug-treated group/average TW of control group.

### Immunoblot analysis

Cells were washed with ice cold PBS (–) twice and lysed with 10 mM Tris-HCl buffer at pH 7.4 containing 50 mM NaCl, 50 mM NaF, 30 mM sodium pyrophosphate, 50 mM Na_3_VO_4_, 5 mM EDTA, aprotinin at 100 Kal U/mL, 1 mM phenylmethylsulfonyl fluoride, 0.5% Nonidet P-40, and 0.1% sodium dodecyl sulfate (SDS). Tumor samples were resected from mice 4 h after the final drug administration, minced with scissors and homogenized by a glass homogenizer with a Teflon pestle in the same buffer described above. The protein concentration of each sample was measured by a BCA Protein Assay Kit (Thermo Fisher Scientific, Waltham, MA). Then, 10 μg of lysate from each cell line or tissue was separated by SDS-polyacrylamide gel electrophoresis, and transferred onto a nitrocellulose membrane. After blocking with 5% skim milk, the membrane was incubated with a primary antibody. Targeted antigens were detected by incubating with anti-phospho AKT (Ser473) antibody, anti-phospho AKT (Thr308) antibody, anti-pan AKT antibody, anti-phospho S6 antibody, anti-S6 antibody, anti-phospho IGF-1R β (Tyr1135/1136) antibody, anti-phospho MEK 1/2 (Ser217/221) antibody, anti-phospho ERK 1/2 (Thr203/Tyr204) antibody, anti-phospho 4E-BP1 (Thr37/46), anti-AKT antibody, anti-S6 antibody, anti-cleaved PARP at Asp214 antibody, anti-PTEN antibody, anti-Id2 antibody, anti-GAPDH antibody (Cell Signaling Technologies, MA, USA), anti-phospho IGF-1R antibody (Abcam, Cambridge, UK) or anti-α tubulin antibody (Sigma-Aldrich, MO, USA), followed by fluorescently tagged secondary antibodies (Thermo Fisher Scientific, CA, USA). Visualization of bound antibody was carried out using fluorescently tagged secondary antibodies (Thermo Fisher Scientific, Waltham, MA) and the signals were detected and digitized by an Odyssey CLx Imaging System (LI-COR Corp., Lincoln, NE). An anti-α tubulin antibody (Sigma-Aldrich, St. Louis, MO) was used to provide data for a loading control.

### Luciferase reporter assay

1 × 10^6^ of cells (RD-ES and A673) or 2.5 × 10^5^ cells (HOS) were seeded into 6 well culture plates and cultured for 24 h. Each cell was co-transfected with 1 μg of pGL3-Id2-2755 and 1 μg of Renilla (*Renilla reniformis*) luciferase internal control plasmid, pGL3-CMV (Promega Corporation, WI, USA) *via* the use of Lipofectamine 2000 (Thermo Fisher Scientific, CA, USA). The culture medium was changed after 6 h of transfection, and cells were passaged to a 96 well plate and treated with ZSTK474 at final concentrations of 0.3 μM, 1 μM, 3 μM or 10 μM. Luciferase activity was detected using the Dual Glo Luciferase Assay System (Promega Corporation, WI, USA). The percentage of luciferase activity was calculated by the luciferase activity of firefly (*Photinus pyralis*) luciferase derived from pGL3-Id2-2755 divided by the Renilla luciferase from pGL3-CMV. pGL3-Id2-2755 was kindly gifted by Dr. Tokino [62].

### Immunohistochemistry

Tumors were resected from mice, fixed with neutral formalin and embedded in paraffin. Four μm sections were cut and deparaffinized in xylene, followed by rehydration through a graded ethanol series (100% to 50%) to PBS (–). After incubation with Dako Real Target Retrieval Solution, pH 6 (Agilent Technologies, Santa Clara, CA) in boiling water bath for 40 min, tumor sections were treated with 3% H_2_O_2_ in PBS (–) and blocked with 10% normal goat serum. Following this, they were incubated with anti-p-S6 antibody, anti-cleaved PARP antibody (Cell Signaling Technologies, MA, USA) or anti-CD31 antibody (Abcam, Cambridge, UK). The bound antibodies were visualized by using EnVision+System HRP labeled polymer antibody and DAB kit (Agilent Technologies, CA, USA), and the counterstaining was performed with hematoxylin. The number of nucleus of CD31-positive cell was counted as vascular endothelial cells.

### Statistical analysis

Data was expressed as mean ± SEM (*in vivo* studies, *n* = 6). Statistical significance of antitumor efficacy was assessed by a Mann–Whitney *U* test or Student's *t* test or Welch *t* test for comparison of two samples on days 10 (MES-SA and MES-SA/Dx5) and days 14 (SJCRH30, SK-UT-1, A673 and SYO-1). The statistical significance of observed anti-angiogenic effects was assessed by a Student's *t* test for comparison of two samples. The statistical significance of aberrant gene status and protein expression levels was determined by either a Mann–Whitney *U* test or Welch *t* test. The statistical significance of luciferase activity was assessed by a Student's *t* test. In all analyses, *P* < 0.05 was considered statistically significant.

## SUPPLEMENTARY MATERIALS FIGURES AND TABLES



## Abbreviations

JFCR: Japanese Foundation for Cancer Research
p-: phosphorylated-
EWSR1: Ewing sarcoma region 1
FLI1: Friend leukemia virus integration 1
PAX3: Paired box gene 3
FOXO1: Forkhead box O1A
SS18: synovial sarcoma translocation: chromosome 18
SSX: synovial sarcoma: X breakpoint
IHC: immunohistochemistry.
