# Supplementary material for: Antitumor profile of the PI3K inhibitor ZSTK474 in human sarcoma cell lines
Source: Oncotarget. 2018 Oct 12;9(80):35141–61. doi: 10.18632/oncotarget.26216 (PMC6205545; doi:10.18632/oncotarget.26216)
Supplement: Supplementary file 1 [file oncotarget-09-35141-s001.pdf]

Antitumor profile of the PI3K inhibitor ZSTK474 in human sarcoma cell lines

SUPPLEMENTARY MATERIALS

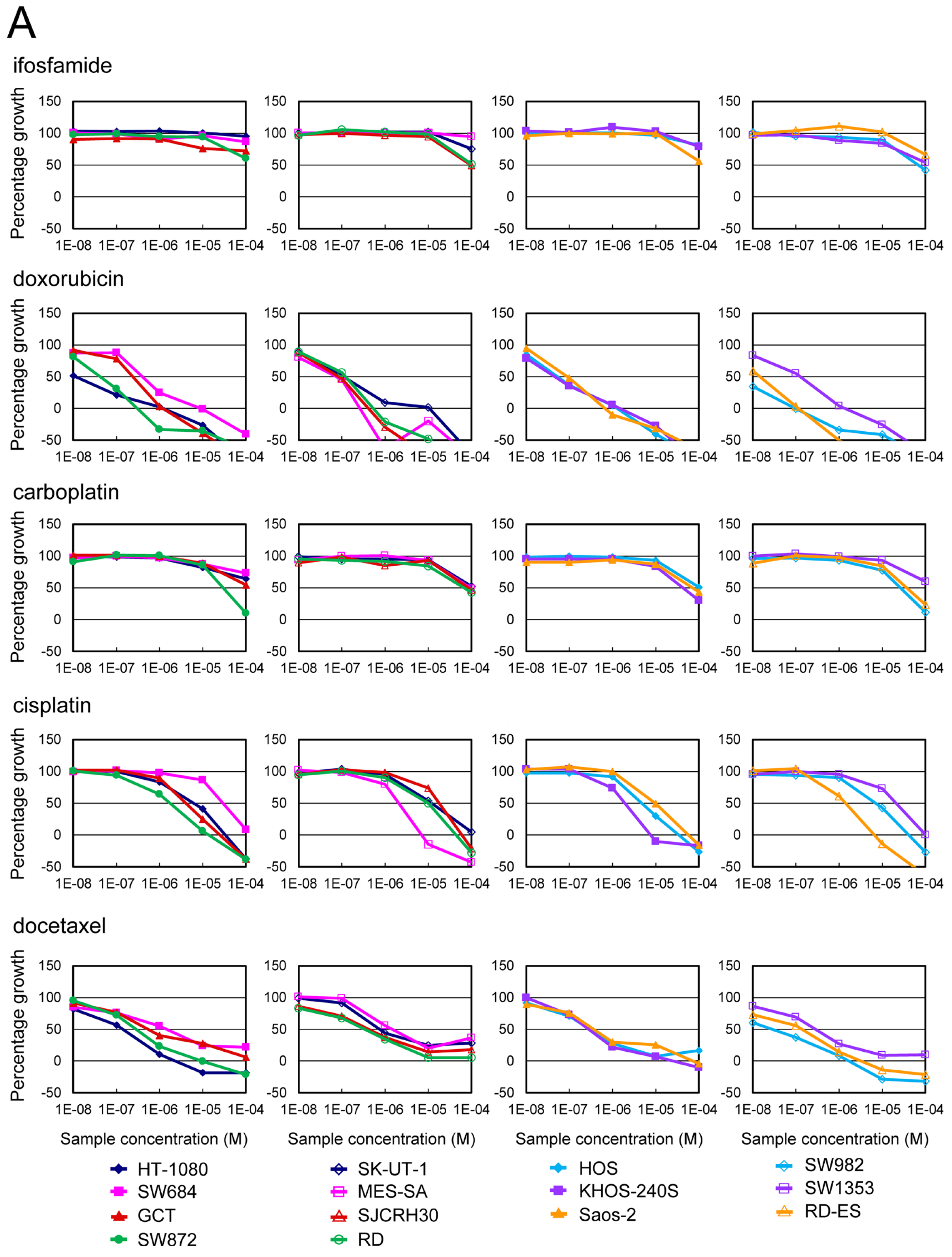

B

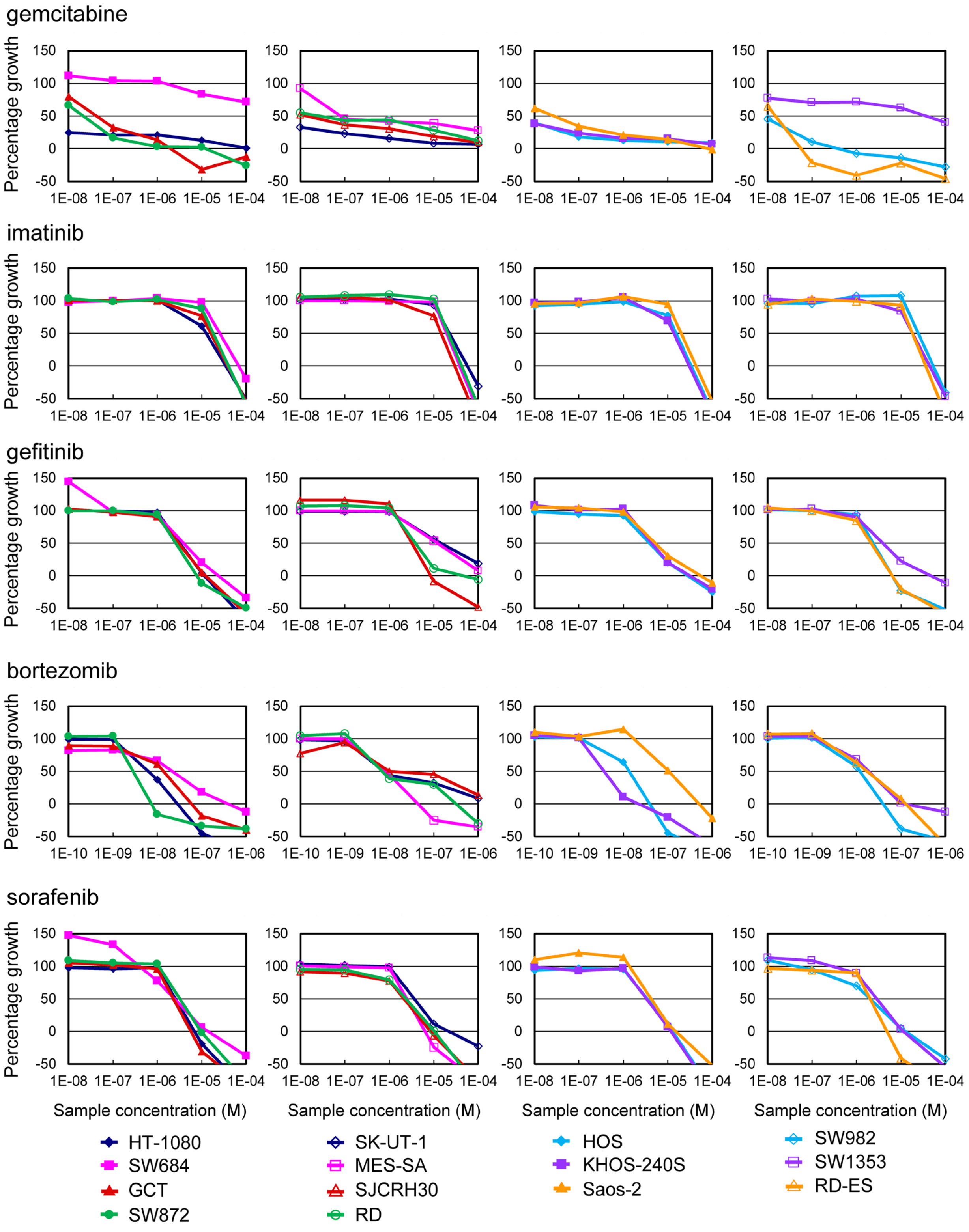
gemcitabine
imatinib
gefitinib
bortezomib
sorafenib
Percentage growth
150
100
50
0
-50
1E-08 1E-07 1E-06 1E-05 1E-04
1E-10 1E-09 1E-08 1E-07 1E-06
Sample concentration (M)
HT-1080
SW684
GCT
SW872
SK-UT-1
MES-SA
SJCRH30
RD
HOS
KHOS-240S
Saos-2
SW982
SW1353
RD-ES

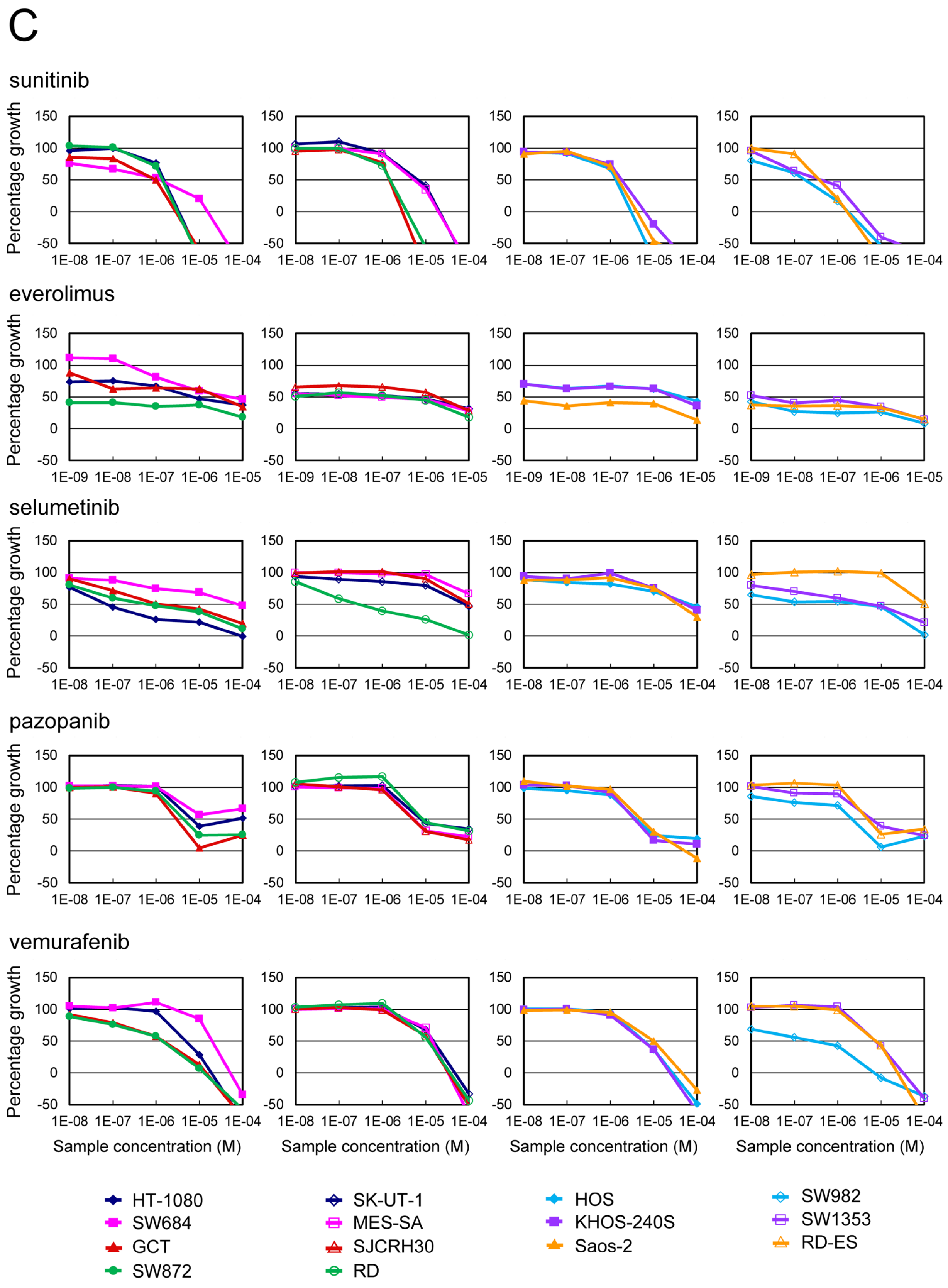

C
sunitinib
everolimus
selumetinib
pazopanib
vemurafenib
Percentage growth
150
100
50
0
-50
1E-08 1E-07 1E-06 1E-05 1E-04
1E-09 1E-08 1E-07 1E-06 1E-05
Sample concentration (M)
HT-1080
SW684
GCT
SW872
SK-UT-1
MES-SA
SJCRH30
RD
HOS
KHOS-240S
Saos-2
SW982
SW1353
RD-ES

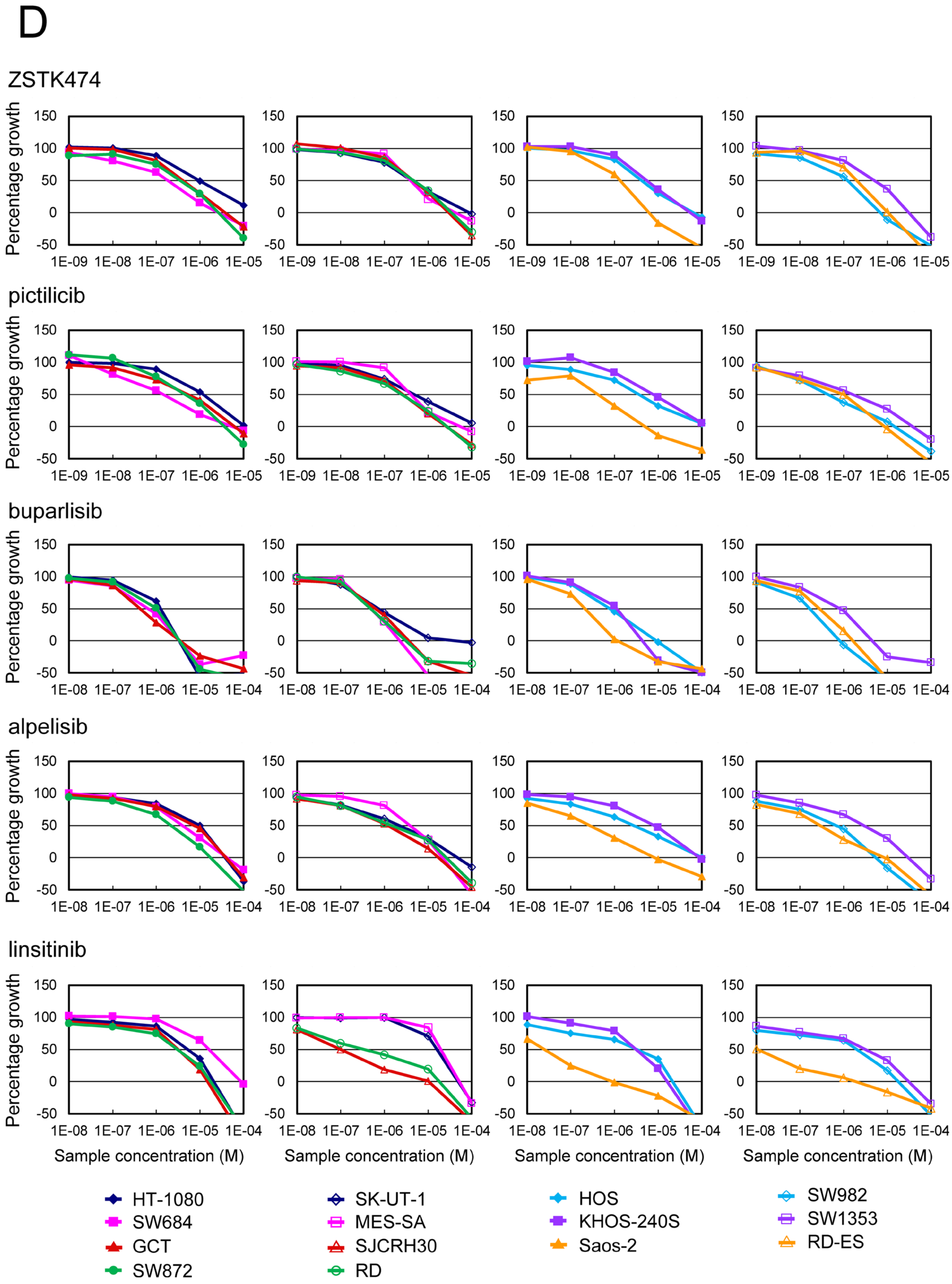
D
ZSTK474
pictilicib
buparlisib
alpelisib
linsitinib
Percentage growth
150
100
50
0
-50
1E-09 1E-08 1E-07 1E-06 1E-05
1E-08 1E-07 1E-06 1E-05 1E-04
Sample concentration (M)
HT-1080
SW684
GCT
SW872
SK-UT-1
MES-SA
SJCRH30
RD
HOS
KHOS-240S
Saos-2
SW982
SW1353
RD-ES

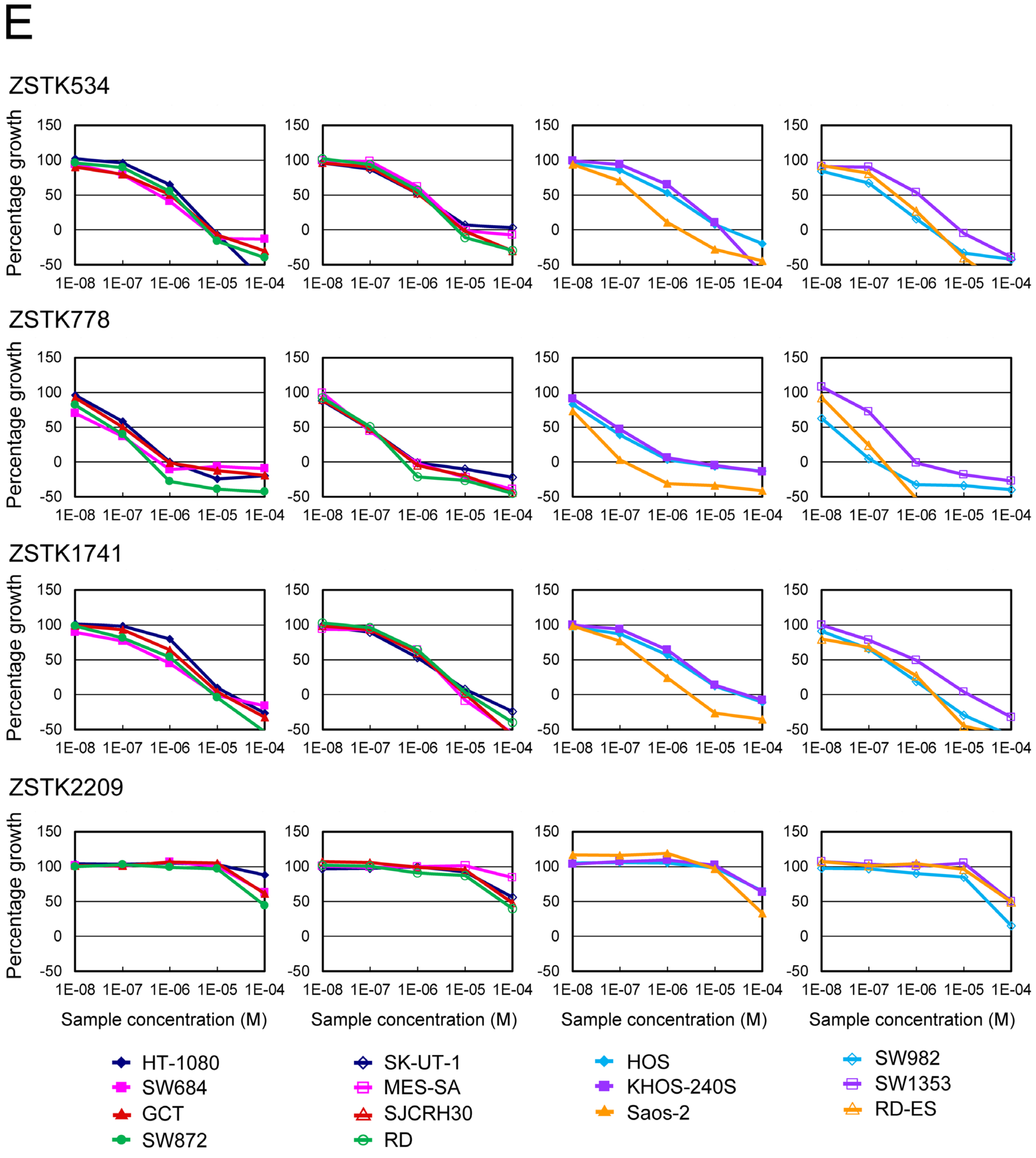

Supplementary Figure 1: Concentration response curves of 24 anticancer agents against 14 sarcoma cell lines. Growth inhibitory activity of each agent against each of the sarcoma cell lines within the panel was assessed as described in the Materials and Methods section. Concentration response curves of ifosfamide, doxorubicin, carboplatin, cisplatin and docetaxel (**A**); gemcitabine, imatinib, gefitinib, bortezomib and sorafenib (**B**); sunitinib, everolimus, selumetinib, pazopanib and vemurafenib (**C**); ZSTK474, pictilicib, buparlisib, alpelisib and linsitinib (**D**); ZSTK534, ZSTK778, ZSTK1741 and ZSTK2209 (**E**). Symbols were indicated as follows: closed blue diamonds, HT-1080; closed pink squares, SW684; closed red triangles, GCT; closed green circles, SW872; open blue diamonds, SK-UT-1; open pink squares, MES-SA; open red triangles, SJCRH30; open green circles, RD; closed cyan diamonds, HOS; closed purple squares, KHOS-240S; closed orange triangles, Saos-2; open cyan diamonds, SW982; open purple squares, SW1353; open orange triangles, RD-ES.

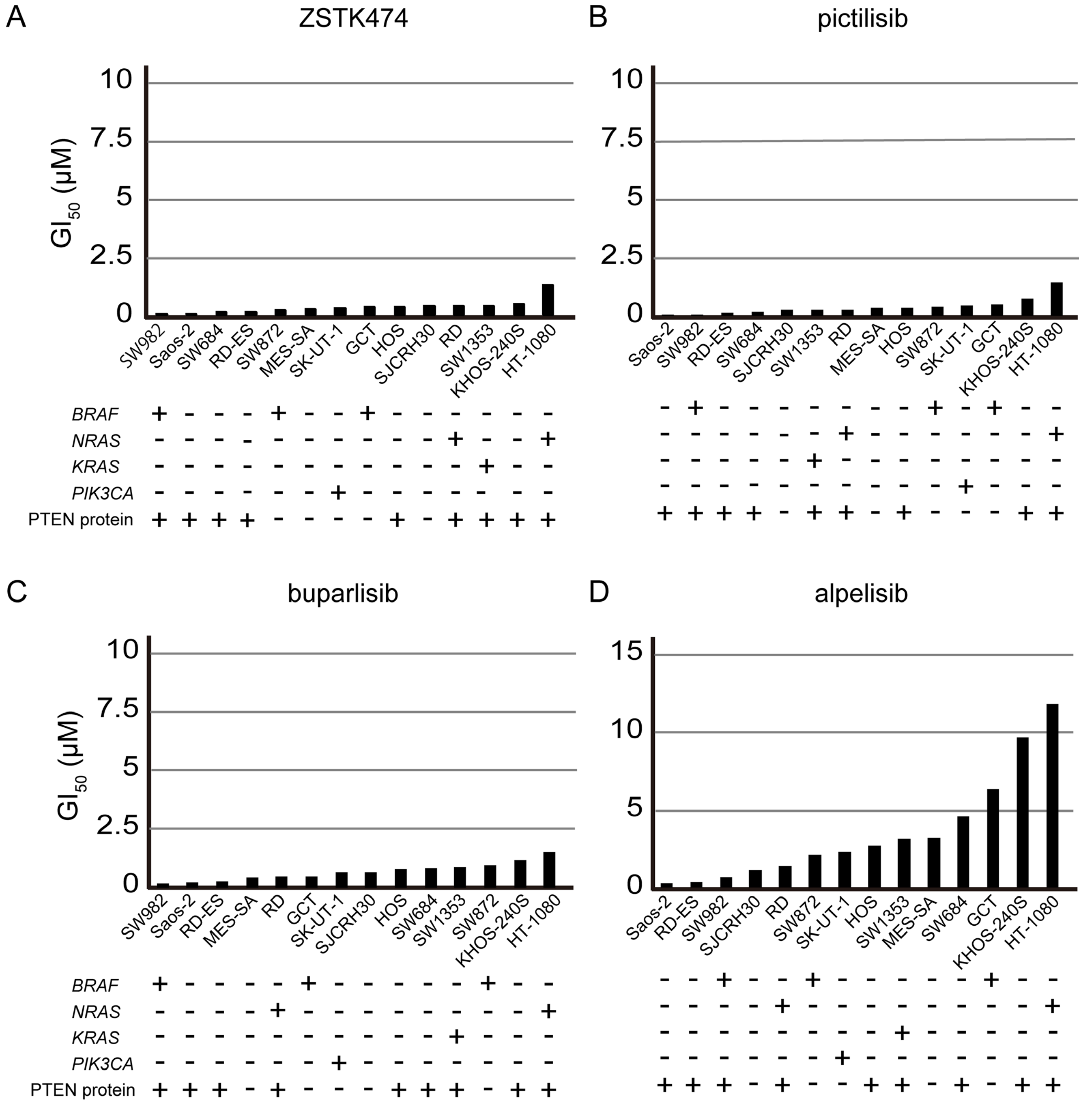

Supplementary Figure 2: Association between drug efficacy of PI3K inhibitors and mutation status of *BRAF*, *KRAS*, *NRAS* and *PIK3CA* or protein expression status of PTEN. (**A–D**) Fourteen sarcoma cell lines were arranged in ascending order of GI50 concentrations of the pan-PI3K inhibitors, ZSTK474 (A), pictilisib (B), buparlisib (C) or α-specific PI3K inhibitor, alpelisib (D), with the mutation statuses of *BRAF*, *NRAS*, *KRAS* and *PIK3CA* and protein expression status of PTEN also indicated. No significant associations between mutation status and the efficacy of PI3K inhibitors were observed.

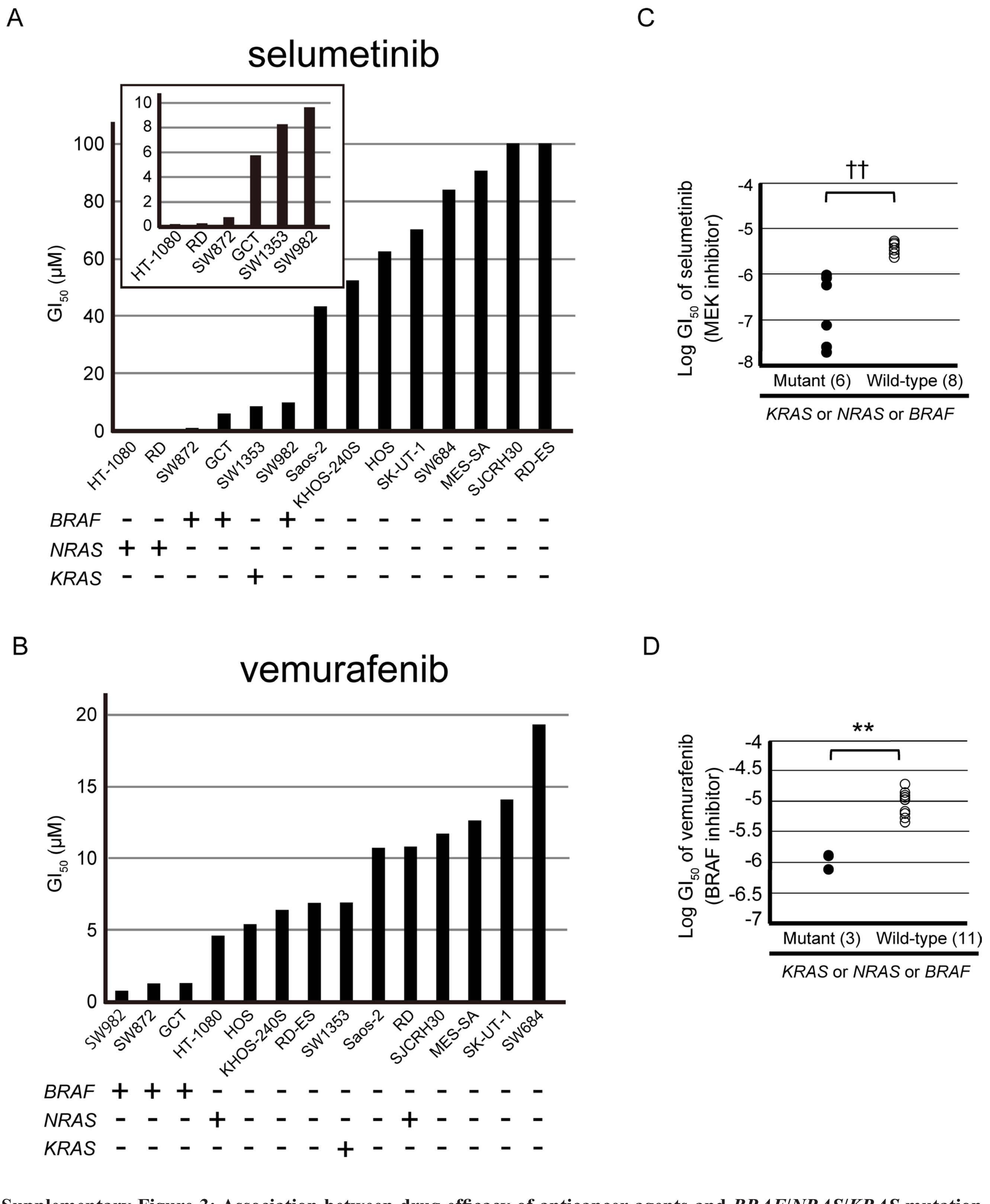

Supplementary Figure 3: Association between drug efficacy of anticancer agents and *BRAF*/*NRAS*/*KRAS* mutation. (**A**, **B**) Fourteen sarcoma cell lines were arranged in ascending order of GI_{50} concentrations of MEK inhibitor (selmetinib) (A) or BRAF inhibitor (vemurafenib) (B), with the mutation statuses of *BRAF*, *NRAS* and *KRAS* genes also indicated. Inserted graph is an enlarged view of first 6 cell lines harboring *BRAF/NRAS/KRAS* mutation (A). (**C**, **D**) Differences in GI_{50} values of selumetinib (C) or vemurafenib (D) between two groups of sarcoma cell lines with or without *BRAF/NRAS/KRAS* mutation. The significance of any differences observed was determined by Mann–Whitney *U* test ($^{**}p < 0.01$)/ Welch *t* test ($^{††}p < 0.01$).

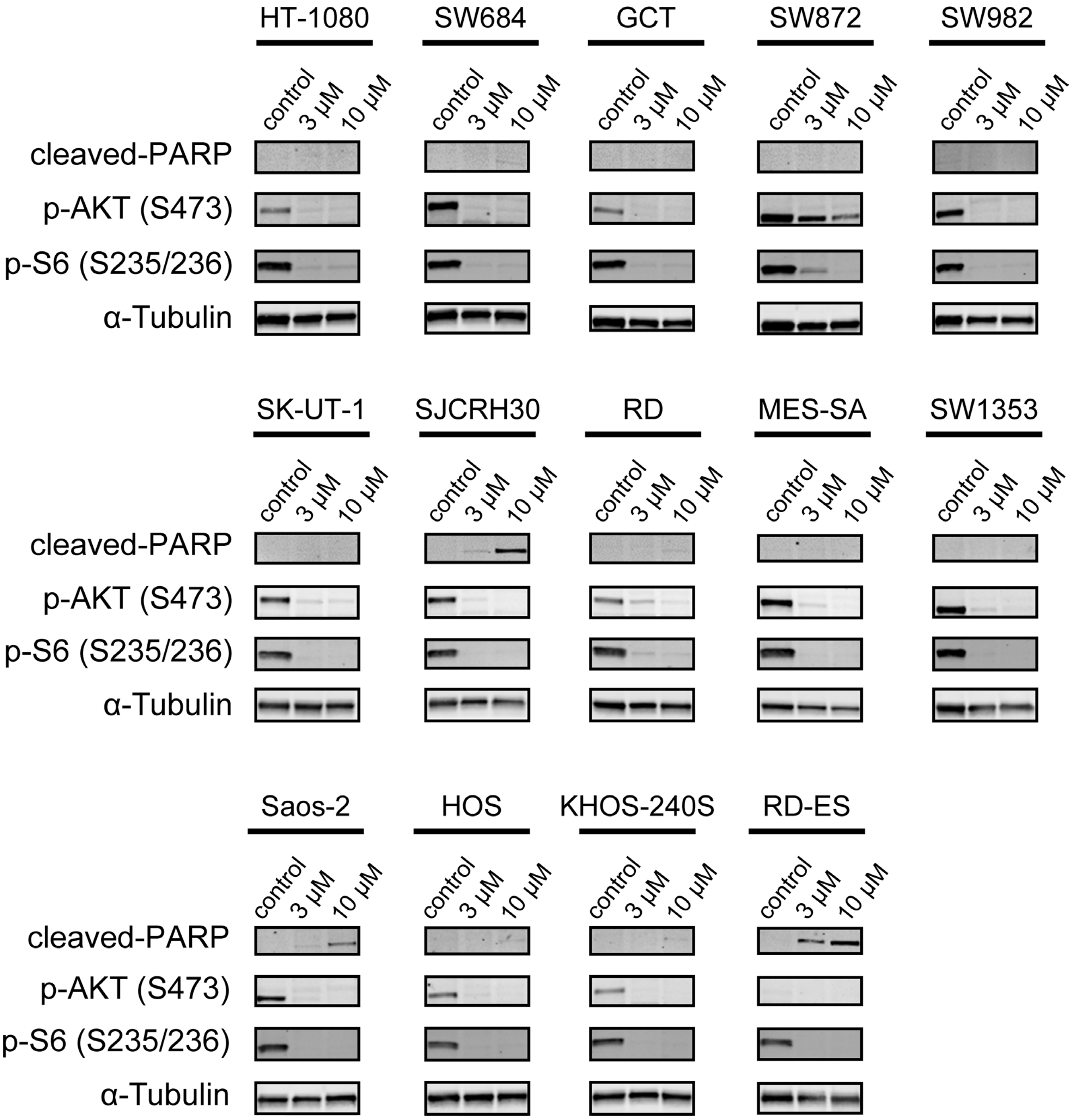

Supplementary Figure 4: Effect of ZSTK474 on PI3K signaling pathway and apoptosis progression in 14 sarcoma cell lines *in vitro*. The effect of ZSTK474 at higher concentrations against sarcoma cells was examined by Western blot analysis. Cells were treated with ZSTK474 at concentrations of 3 μM and 10 μM for 48 h and cell lysates were immunoblotted to detect phosphorylated AKT (Ser473), phosphorylated S6 (Ser235/236) and α-tubulin as described in the Materials and Methods section.

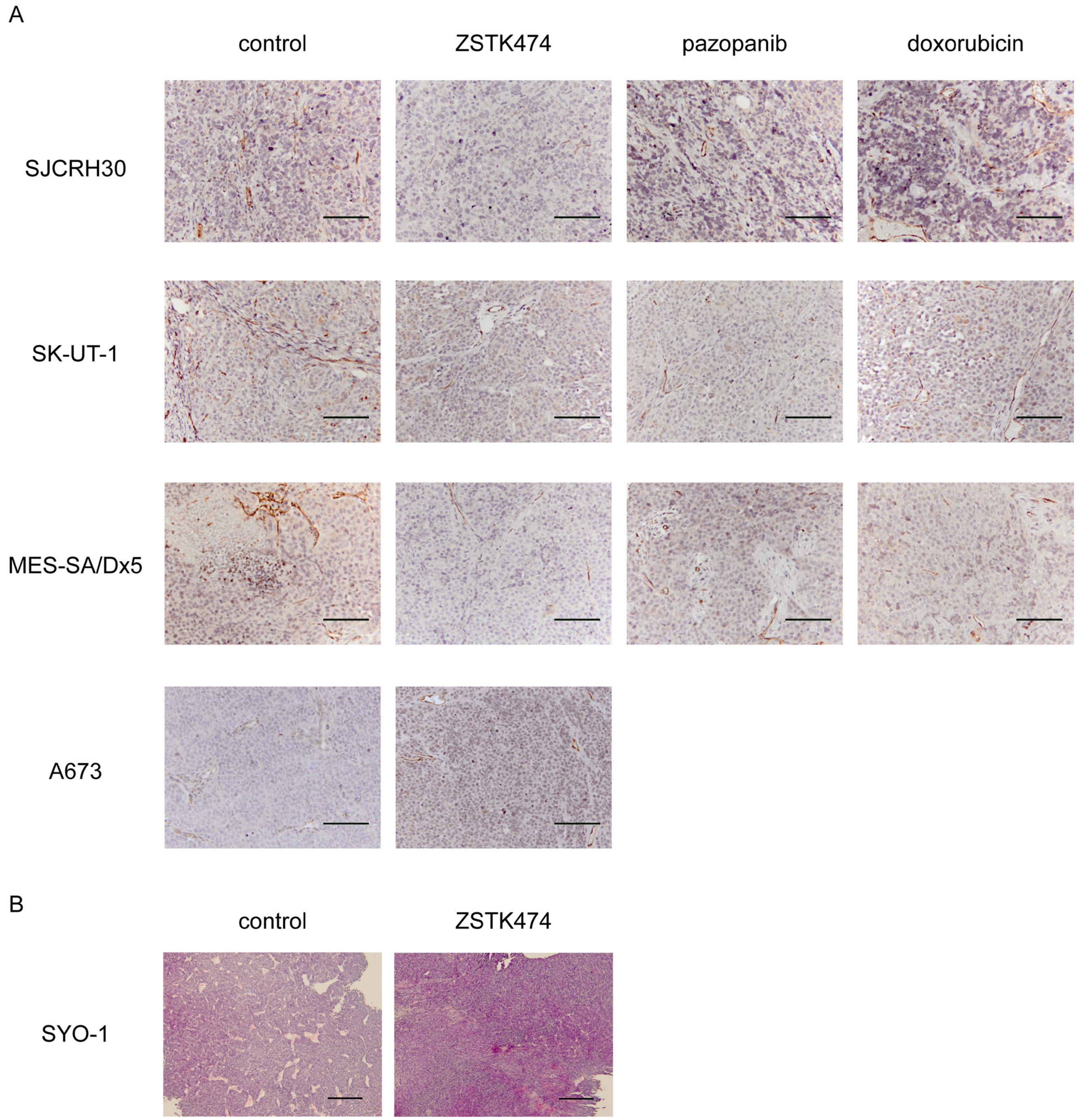

Supplementary Figure 5: Representative images of xenografted tumor tissue sections of SJCRH30, SK-UT-1, MES-SA/Dx5, A673 and SYO-1 resected from mice before and after administration of ZSTK474, pazopanib or doxorubicin. (**A**) Representative images of tumor vascularization in a tumor tissue section derived from SJCRH30, SK-UT-1 or MES-SA/Dx5 resected from control mice or mice administered with ZSTK474, pazopanib and doxorubicin, and an A673 tumor tissue section resected from control mice or mice administrated with ZSTK474, respectively. Vascular endothelial cells were detected by immunohistochemistry using an anti-CD31 antibody. Scale bars: 100 µm. (**B**) Hematoxylin-eosin staining images of a SYO-1 tumor tissue section resected from control mice or mice treated with ZSTK474. Scale bars: 100 µm.

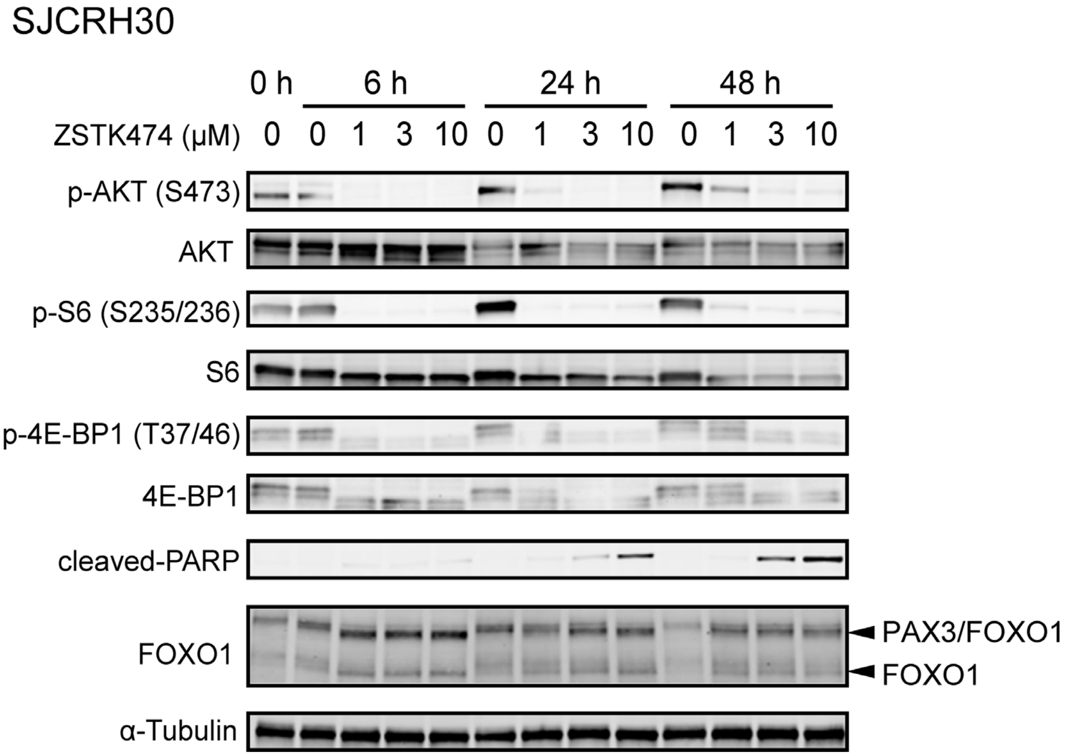

Supplementary Figure 6: Effect on PI3K-downstream signaling pathway, apoptosis progression and protein expression of PAX3-FOXO1 upon treatment with ZSTK474 in alveolar rhabdomyosarcoma cell line, SJCRH30. SJCRH30 cells were treated with ZSTK474 at the specified concentrations for the indicated time. Western blot analysis of phosphorylated AKT (S473), total AKT, phosphorylated S6 (S235/236), total S6, phosphorylated 4E-BP1 (T37/46), total 4E-BP1, cleaved-PARP, FOXO1 and α-tubulin were performed as described in the Materials and Methods section.

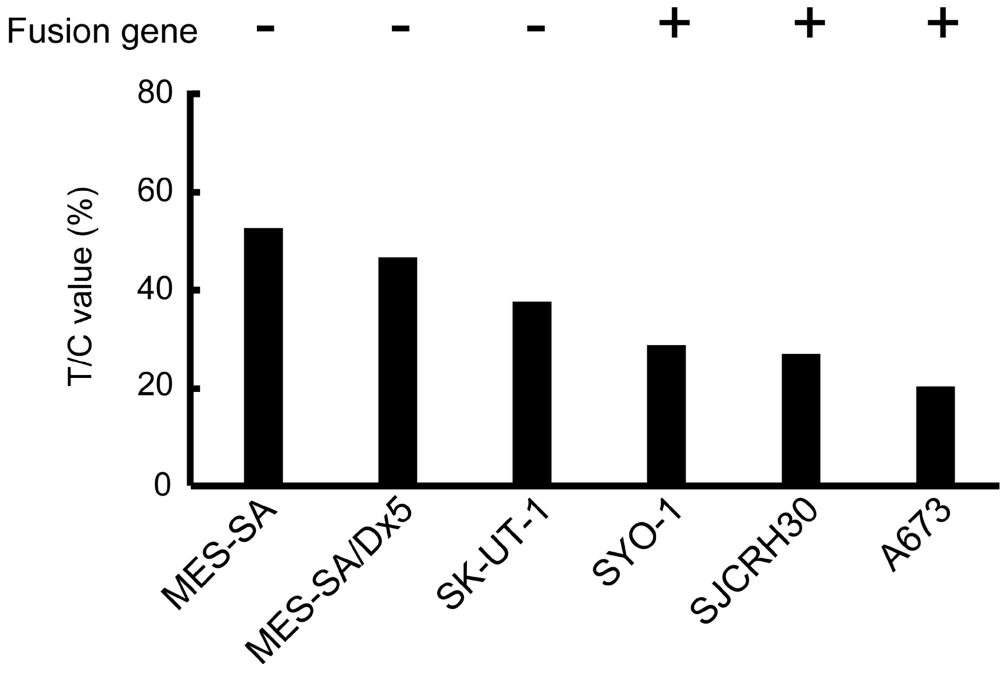

Supplementary Figure 7: *In vivo* efficacy of ZSTK474 toward sarcoma cells xenografted in nude mice. The *in vivo* efficacy of ZSTK474 against sarcoma cells was evaluated by treated/control ratio (T/C)%. “Fusion genes” included the following genes: *EWSR1-FLI1* (A673), *SS18-SX2* (SYO-1) and *PAX3-FOXO1* (SJCRH30).

Supplementary Table 1: GI_{50} concentrations (M) for 24 anticancer agents against 14 sarcoma cell lines

compounds		HT-1080	SW684	GCT	SK-UT-1	SJCRH30	RD	HOS	KHOS-240S	Saos-2	SW872	SW982	SW1353	MES-SA	RD-ES
ZSTK474	assay-1	9.43.E-07	1.85.E-07	4.12.E-07	4.22.E-07	4.62.E-07	4.69.E-07	4.15.E-07	5.50.E-07	1.36.E-07	3.50.E-07	1.23.E-07	5.00.E-07	3.99.E-07	1.98.E-07
	assay-2	1.84.E-06	2.40.E-07	4.48.E-07	3.62.E-07	4.41.E-07	4.52.E-07	4.58.E-07	5.48.E-07	1.45.E-07	2.82.E-07	9.69.E-08	4.52.E-07	2.91.E-07	2.30.E-07
ZSTK778	assay-1	1.37.E-07	3.99.E-08	9.77.E-08	8.27.E-08	9.16.E-08	1.04.E-07	5.62.E-08	8.54.E-08	2.15.E-08	5.86.E-08	1.69.E-08	1.98.E-07	7.71.E-08	4.12.E-08
	assay-2	1.48.E-07	3.91.E-08	1.21.E-07	4.20.E-08	4.67.E-08	7.32.E-08	5.71.E-08	5.92.E-08	1.43.E-08	3.66.E-08	1.20.E-08	6.71.E-08	3.00.E-08	2.39.E-08
ZSTK534	assay-1	1.64.E-06	5.76.E-07	1.06.E-06	1.10.E-06	1.11.E-06	1.27.E-06	1.17.E-06	1.88.E-06	2.17.E-07	1.16.E-06	2.11.E-07	1.18.E-06	1.54.E-06	3.75.E-07
	assay-2	1.99.E-06	5.14.E-07	1.02.E-06	9.32.E-07	9.69.E-07	9.72.E-07	9.98.E-07	1.66.E-06	1.64.E-07	8.16.E-07	2.35.E-07	1.11.E-06	5.99.E-07	3.48.E-07
ZSTK1741	assay-1	2.66.E-06	6.95.E-07	1.74.E-06	1.16.E-06	1.49.E-06	1.70.E-06	1.46.E-06	1.92.E-06	3.23.E-07	1.19.E-06	2.23.E-07	9.13.E-07	1.55.E-06	2.78.E-07
	assay-2	3.56.E-06	1.46.E-06	1.51.E-06	8.41.E-07	1.55.E-06	1.51.E-06	1.30.E-06	2.44.E-06	1.87.E-06	1.16.E-06	2.43.E-07	1.65.E-06	7.03.E-07	3.48.E-07
ZSTK2209	assay-1	1.00.E-04	1.00.E-04	1.00.E-04	1.00.E-04	9.03.E-05	6.03.E-05	1.00.E-04	1.00.E-04	5.45.E-05	7.82.E-05	3.20.E-05	9.95.E-05	1.00.E-04	9.50.E-05
	assay-2	1.00.E-04	1.00.E-04	1.00.E-04	1.00.E-04	9.02.E-05	6.50.E-05	1.00.E-04	1.00.E-04	5.26.E-05	6.15.E-05	3.24.E-05	9.61.E-05	1.00.E-04	7.10.E-05
pictilisib	assay-1	1.17.E-06	1.43.E-07	5.31.E-07	4.84.E-07	2.62.E-07	2.52.E-07	3.61.E-07	7.66.E-07	4.19.E-08	4.62.E-07	4.40.E-08	1.62.E-07	4.03.E-07	9.74.E-08
	assay-2	1.76.E-06	2.05.E-07	4.56.E-07	4.02.E-07	2.85.E-07	2.98.E-07	3.84.E-07	7.64.E-07	4.11.E-08	3.45.E-07	9.65.E-08	3.86.E-07	3.26.E-07	1.66.E-07
buparlisib	assay-1	1.26.E-06	6.44.E-07	4.25.E-07	7.25.E-07	6.28.E-07	4.91.E-07	7.84.E-07	1.13.E-06	2.12.E-07	1.02.E-06	1.67.E-07	8.39.E-07	4.89.E-07	2.81.E-07
	assay-2	1.76.E-06	1.06.E-06	5.87.E-07	5.99.E-07	7.21.E-07	4.48.E-07	7.82.E-07	1.20.E-06	2.33.E-07	8.88.E-07	2.26.E-07	9.23.E-07	3.80.E-07	2.73.E-07
alpelisib	assay-1	9.89.E-06	3.97.E-06	7.49.E-06	2.17.E-06	1.18.E-06	1.48.E-06	2.73.E-06	8.26.E-06	2.78.E-07	2.15.E-06	6.77.E-07	2.90.E-06	3.85.E-06	2.94.E-07
	assay-2	1.35.E-05	5.08.E-06	5.05.E-06	2.31.E-06	9.74.E-07	1.20.E-06	2.54.E-06	1.09.E-05	2.24.E-07	1.96.E-06	5.55.E-07	3.21.E-06	2.48.E-06	3.21.E-07
everolimus	assay-1	7.11.E-07	5.38.E-06	2.84.E-06	2.97.E-07	1.79.E-06	1.96.E-07	4.53.E-06	2.96.E-06	1.00.E-09	1.00.E-09	1.00.E-09	1.47.E-09	5.32.E-08	1.00.E-09
	assay-2	1.00.E-05	5.54.E-06	3.37.E-06	1.00.E-09	2.41.E-06	2.08.E-07	3.66.E-06	1.91.E-06	1.00.E-09	1.00.E-09	1.00.E-09	2.23.E-08	2.30.E-09	6.80.E-09
linsitinib	assay-1	5.25.E-06	1.61.E-05	3.15.E-06	1.58.E-05	1.03.E-07	3.50.E-07	3.25.E-06	3.10.E-06	2.50.E-08	3.10.E-06	2.03.E-06	3.15.E-06	1.94.E-05	1.07.E-08
	assay-2	5.98.E-06	1.31.E-05	2.47.E-06	1.23.E-05	1.49.E-07	4.35.E-07	2.69.E-06	2.76.E-06	2.35.E-08	2.93.E-06	1.79.E-06	3.14.E-06	1.43.E-05	1.00.E-08
selumetinib	assay-1	7.35.E-08	7.72.E-05	1.22.E-06	8.29.E-05	1.00.E-04	2.86.E-07	6.89.E-05	5.32.E-05	3.60.E-05	6.63.E-07	3.53.E-06	6.12.E-06	1.00.E-04	1.00.E-04
	assay-2	3.16.E-07	9.05.E-05	1.03.E-05	5.72.E-05	1.00.E-04	2.25.E-07	5.57.E-05	5.13.E-05	5.03.E-05	8.63.E-07	1.58.E-05	1.04.E-05	8.09.E-05	1.00.E-04
vemurafenib	assay-1	4.84.E-06	1.96.E-05	1.46.E-06	1.51.E-05	1.19.E-05	1.16.E-05	5.89.E-06	5.74.E-06	1.01.E-05	1.40.E-06	2.76.E-07	7.44.E-06	1.41.E-05	7.64.E-06
	assay-2	4.38.E-06	1.91.E-05	1.15.E-06	1.31.E-05	1.15.E-05	1.01.E-05	4.93.E-06	7.06.E-06	1.14.E-05	1.15.E-06	1.26.E-06	6.40.E-06	1.11.E-05	6.13.E-06
gefitinib	assay-1	3.23.E-06	3.90.E-06	3.02.E-06	1.43.E-05	3.19.E-06	3.83.E-06	3.89.E-06	4.39.E-06	5.22.E-06	2.61.E-06	2.39.E-06	3.94.E-06	1.22.E-05	2.14.E-06
	assay-2	3.49.E-06	4.15.E-06	3.40.E-06	6.95.E-06	2.67.E-06	4.15.E-06	3.59.E-06	3.91.E-06	5.40.E-06	2.72.E-06	2.54.E-06	4.03.E-06	4.90.E-06	2.38.E-06
sorafenib	assay-1	2.56.E-06	2.43.E-06	2.30.E-06	3.60.E-06	2.14.E-06	2.36.E-06	3.42.E-06	3.32.E-06	4.23.E-06	3.22.E-06	2.04.E-06	2.85.E-06	2.44.E-06	2.02.E-06
	assay-2	2.76.E-06	3.98.E-06	2.39.E-06	3.23.E-06	2.65.E-06	2.70.E-06	3.19.E-06	3.10.E-06	5.07.E-06	2.83.E-06	3.26.E-06	3.49.E-06	2.20.E-06	2.27.E-06
imatinib	assay-1	1.26.E-05	2.53.E-05	1.61.E-05	2.25.E-05	1.46.E-05	2.09.E-05	1.54.E-05	1.36.E-05	1.99.E-05	1.82.E-05	2.46.E-05	1.83.E-05	1.93.E-05	1.81.E-05
	assay-2	1.91.E-05	2.56.E-05	1.74.E-05	2.09.E-05	1.39.E-05	1.95.E-05	1.54.E-05	1.41.E-05	2.16.E-05	1.52.E-05	2.25.E-05	1.88.E-05	1.68.E-05	1.77.E-05
sunitinib	assay-1	1.52.E-06	1.24.E-06	9.79.E-07	6.76.E-06	1.46.E-06	1.51.E-06	1.34.E-06	1.83.E-06	1.52.E-06	1.42.E-06	1.80.E-07	4.13.E-07	5.46.E-06	3.78.E-07
	assay-2	1.98.E-06	4.18.E-06	1.22.E-06	2.75.E-06	1.52.E-06	1.70.E-06	1.36.E-06	1.79.E-06	1.56.E-06	1.17.E-06	3.74.E-07	1.42.E-06	1.80.E-06	3.11.E-07
pazopanib	assay-1	6.68.E-06	1.00.E-04	2.96.E-06	7.76.E-06	5.05.E-06	8.40.E-06	3.93.E-06	3.59.E-06	5.00.E-06	4.34.E-06	2.14.E-06	6.04.E-06	5.26.E-06	4.93.E-06
	assay-2	3.16.E-07	9.05.E-05	1.03.E-05	6.41.E-06	5.39.E-06	9.31.E-06	3.81.E-06	3.08.E-06	4.94.E-06	4.09.E-06	2.45.E-06	6.03.E-06	3.85.E-06	3.83.E-06
bortezomib	assay-1	6.32.E-09	2.17.E-08	1.39.E-08	7.71.E-09	1.14.E-08	6.95.E-09	1.35.E-08	3.68.E-09	1.05.E-07	2.83.E-09	1.21.E-08	1.87.E-08	8.03.E-09	1.76.E-08
	assay-2	1.03.E-08	4.75.E-08	2.24.E-08	6.01.E-09	6.37.E-09	4.70.E-09	6.22.E-09	3.94.E-09	1.83.E-07	2.97.E-09	1.01.E-08	1.69.E-08	3.75.E-09	9.13.E-09
doxorubicin	assay-1	1.11.E-08	4.01.E-07	2.38.E-07	1.10.E-07	8.36.E-08	1.23.E-07	5.27.E-08	4.63.E-08	8.90.E-08	4.11.E-08	1.00.E-08	1.28.E-07	7.55.E-08	1.46.E-08
	assay-2	1.42.E-08	3.22.E-07	1.64.E-07	2.51.E-08	2.13.E-08	4.56.E-08	2.29.E-08	1.73.E-08	4.17.E-08	1.97.E-08	1.00.E-08	3.05.E-08	1.00.E-08	1.52.E-08
docetaxel	assay-1	1.40.E-09	1.46.E-08	5.52.E-09	7.59.E-09	4.22.E-09	3.36.E-09	3.05.E-09	2.83.E-09	3.73.E-09	2.87.E-09	2.90.E-10	2.85.E-09	1.49.E-08	1.38.E-09
	assay-2	4.33.E-09	3.71.E-08	1.19.E-08	6.32.E-09	5.46.E-09	3.42.E-09	3.93.E-09	2.77.E-09	6.92.E-09	3.92.E-09	6.09.E-10	3.83.E-09	4.85.E-09	2.06.E-09
gemcitabine	assay-1	1.00.E-08	1.00.E-04	4.25.E-08	1.00.E-08	1.41.E-08	2.73.E-08	1.00.E-08	1.00.E-08	2.72.E-08	2.12.E-08	1.00.E-08	3.73.E-05	7.97.E-08	1.49.E-08
	assay-2	1.00.E-08	1.00.E-04	5.91.E-08	1.00.E-08	1.00.E-08	6.31.E-08	1.00.E-08	1.00.E-08	6.69.E-08	1.98.E-08	1.00.E-08	3.61.E-05	1.00.E-08	1.47.E-08
cisplatin	assay-1	6.22.E-06	2.93.E-05	4.07.E-06	1.20.E-05	1.77.E-05	9.89.E-06	4.75.E-06	1.91.E-06	9.72.E-06	1.76.E-06	6.92.E-06	2.07.E-05	2.06.E-06	1.42.E-06
	assay-2	1.07.E-05	3.05.E-05	8.47.E-06	9.00.E-06	1.78.E-05	9.22.E-06	3.51.E-06	1.47.E-06	1.02.E-05	2.20.E-06	6.81.E-06	2.04.E-05	9.99.E-07	1.34.E-06
carboplatin	assay-1	1.00.E-04	1.00.E-04	1.00.E-04	1.00.E-04	8.26.E-05	6.65.E-05	1.00.E-04	4.23.E-05	7.11.E-05	2.94.E-05	2.62.E-05	1.00.E-04	9.30.E-05	3.60.E-05
	assay-2	1.00.E-04	1.00.E-04	1.00.E-04	1.00.E-04	9.28.E-05	8.86.E-05	1.00.E-04	5.58.E-05	8.07.E-05	3.49.E-05	3.16.E-05	1.00.E-04	2.91.E-05	3.85.E-05
ifosfamide	assay-1	1.00.E-04	1.00.E-04	1.00.E-04	1.00.E-04	9.71.E-05	1.00.E-04	1.00.E-04	1.00.E-04	1.00.E-04	1.00.E-04	6.70.E-05	1.00.E-04	1.00.E-04	1.00.E-04
	assay-2	1.00.E-04	1.00.E-04	1.00.E-04	1.00.E-04	9.90.E-05	1.00.E-04	1.00.E-04	1.00.E-04	1.00.E-04	1.00.E-04	6.46.E-05	1.00.E-04	1.00.E-04	1.00.E-04

Supplementary Table 2: Result of COMPARE analysis using ZSTK474 as a seed

Rank	r	Chemical name	*p* value
1	0.939	ZSTK474	<0.00001
2	0.921	ZSTK778	<0.00001
3	0.907	ZSTK534	<0.00001
4	0.888	pictilisib	<0.00001
5	0.867	ZSTK1741	0.00003
6	0.852	ZSTK778	0.00006
7	0.835	ZSTK534	0.0001
8	0.811	pictilisib	0.0002
9	0.808	buparlisib	0.0003
10	0.801	buparlisib	0.0003
11	0.763	ZSTK1741	0.0009
12	0.696	everolimus	0.004
13	0.676	ZSTK2209	0.006
14	0.635	ZSTK2209	0.011
15	0.628	alpelisib	0.012
16	0.624	alpelisib	0.013
17	0.609	everolimus	0.016
18	0.47	ifosfamide	0.077
19	0.464	ifosfamide	0.081
20	0.38	carboplatin	0.162
21	0.37	carboplatin	0.174
22	0.253	vemurafenib	0.362
23	0.219	pazopanib	0.432
24	0.18	linsitinib	0.522
25	0.174	sunitinib	0.536
26	0.154	linsitinib	0.584
27	0.13	sunitinib	0.644
28	0.114	docetaxel	0.686
29	0.072	vemurafenib	0.799
30	0.027	pazopanib	0.925
31	0.025	sorafenib	0.929
32	0.001	gefitinib	0.997
33	–0.03	cisplatin	0.914
34	–0.031	gefitinib	0.912
35	–0.106	docetaxel	0.706
36	–0.138	cisplatin	0.624
37	–0.178	doxorubicin	0.527
38	–0.206	doxorubicin	0.462
39	–0.223	gemcitabine	0.425
40	–0.225	gemcitabine	0.420
41	–0.227	bortezomib	0.416
42	–0.284	sorafenib	0.304
43	–0.448	imatinib	0.094
44	–0.458	selumetinib	0.086
45	–0.458	bortezomib	0.086
46	–0.487	selumetinib	0.066
47	–0.784	imatinib	0.0005

Supplementary Table 3: Correlation between drug efficacy and the expression level of signal related proteins

	p-AKT (S473)		p-AKT (T308)		p-S6 (S235/236)		p-IGF-1R (Y1135/1136)		IGF-1R		p-ERK1/2 (T202/Y204)		p-MEK1/2 (S217/221)	
	**r**	***p***	**r**	***p***	**r**	***p***	**r**	***p***	**r**	***p***	**r**	***p***	**r**	***p***
ZSTK474	0.18	0.54	0.20	0.48	0.05	0.85	0.21	0.47	0.15	0.60	0.32	0.26	0.00	0.99
ZSTK778	0.13	0.67	0.12	0.68	–0.05	0.87	0.25	0.38	0.17	0.56	0.24	0.40	–0.09	0.77
ZSTK534	–0.08	0.78	0.01	0.98	–0.18	0.55	0.29	0.31	0.09	0.76	0.22	0.46	–0.12	0.67
ZSTK1741	0.02	0.93	0.04	0.90	–0.04	0.90	0.41	0.15	0.10	0.74	0.19	0.52	–0.05	0.85
ZSTK2209	–0.19	0.52	–0.24	0.41	0.04	0.90	0.30	0.30	0.36	0.20	0.24	0.40	0.37	0.20
pictilisib	0.03	0.91	0.07	0.81	–0.18	0.54	0.33	0.26	0.21	0.46	0.18	0.55	–0.11	0.71
buparlisib	–0.16	0.59	–0.10	0.74	–0.07	0.82	0.41	0.14	0.05	0.88	–0.07	0.82	0.02	0.95
alpelisib	–0.44	0.11	–0.18	0.53	–0.11	0.70	0.55	0.04	0.41	0.15	–0.09	0.76	–0.10	0.74
everolimus	0.02	0.94	–0.11	0.71	0.22	0.45	0.17	0.56	0.37	0.20	–0.12	0.67	0.03	0.91
lisitinib	–0.44	0.11	–0.48	0.09	–0.45	0.10	0.63	0.02	0.44	0.12	–0.39	0.17	–0.10	0.74
selumetinib	–0.32	0.26	–0.33	0.24	0.26	0.37	0.06	0.85	0.16	0.58	0.13	0.65	0.66	0.01
vemurafenib	–0.45	0.11	–0.33	0.25	0.51	0.07	–0.17	0.55	0.30	0.29	0.19	0.52	0.55	0.04
gefitinib	–0.32	0.26	–0.37	0.19	0.01	0.99	0.34	0.24	0.54	0.05	0.28	0.34	0.38	0.18
sorafenib	0.00	1.00	0.04	0.90	0.22	0.46	0.31	0.28	0.00	0.70	–0.03	0.96	0.28	0.34
imatinib	–0.21	0.48	–0.43	0.12	0.05	0.85	–0.12	0.69	0.35	0.23	–0.57	0.03	–0.03	0.91
sunitinib	–0.46	0.10	–0.48	0.08	–0.09	0.75	0.42	0.13	0.37	0.19	0.08	0.79	0.19	0.51
pazopanib	–0.45	0.11	–0.70	0.01	0.07	0.82	0.03	0.91	0.33	0.25	–0.64	0.01	0.01	0.99
bortezomib	0.19	0.51	–0.03	0.92	0.37	0.20	0.04	0.90	0.22	0.45	–0.11	0.70	0.38	0.18
doxorubicin	–0.31	0.28	–0.46	0.10	–0.11	0.70	0.28	0.33	0.26	0.37	–0.34	0.24	–0.10	0.75
docetaxel	–0.50	0.07	–0.63	0.01	–0.16	0.59	0.37	0.19	0.38	0.18	–0.16	0.60	0.18	0.55
gemcitabine	–0.37	0.19	–0.57	0.03	–0.01	0.98	0.22	0.45	0.22	0.46	–0.54	0.05	0.14	0.64
cisplatin	–0.03	0.91	–0.15	0.60	0.34	0.23	0.21	0.48	0.10	0.74	–0.38	0.18	0.11	0.70
carboplatin	0.02	0.95	–0.11	0.72	0.29	0.32	0.29	0.31	0.42	0.13	0.10	0.75	0.30	0.30
ifosfamide	–0.11	0.71	–0.11	0.71	0.05	0.86	0.06	0.84	0.11	0.71	0.38	0.18	0.21	0.47
